# Effectiveness and cost-effectiveness of risk-adapted colorectal cancer screening: a randomized controlled trial and modeling analysis

**DOI:** 10.1186/s40779-025-00671-7

**Published:** 2025-11-24

**Authors:** Hong-Da Chen, Bin Lu, Ju-Fang Shi, Yue-Yang Zhou, Ling-Bin Du, Xian-Zhen Liao, Dong-Hua Wei, Dong Dong, Yi Gao, Chen Zhu, Rong-Biao Ying, Wei-Fang Zheng, Shi-Peng Yan, Hai-Fan Xiao, Juan Zhang, Yun-Xin Kong, Fu-Rong Li, Na Li, Jia-Hui Luo, Chen-Yu Luo, Hermann Brenner, Min Dai

**Affiliations:** 1 Center for Prevention and Early Intervention, National Infrastructures for Translational Medicine, Institute of Clinical Medicine, Peking Union Medical College Hospital, Chinese Academy of Medical Sciences and Peking Union Medical College, Beijing, 100730 China; 2 Department of Cancer Epidemiology, National Cancer Center, National Clinical Research Center for Cancer, Cancer Hospital, Chinese Academy of Medical Sciences and Peking Union Medical College, Beijing, 100021 China; 3 Office of Cancer Screening, National Cancer Center, National Clinical Research Center for Cancer, Cancer Hospital, Chinese Academy of Medical Sciences and Peking Union Medical College, Beijing, 100021 China; 4 Department of Cancer Prevention, Institute of Cancer and Basic Medicine (ICBM), Chinese Academy of Sciences, Cancer Hospital of University of Chinese Academy of Sciences, Zhejiang Cancer Hospital, Hangzhou, 310022 China; 5 Department of Cancer Prevention, Hunan Cancer Hospital, Changsha, 410013 China; 6 Department of Cancer Prevention, Anhui Provincial Cancer Hospital, Hefei, 230031 China; 7 Office of Cancer Prevention and Treatment, Xuzhou Cancer Hospital, Xuzhou, 221005 Jiangsu China; 8 Department of Colorectum Surgery, Tumor Hospital of Yunnan Province, Third Affiliated Hospital of Kunming Medical University, Kunming, 650118 China; 9 Department of Surgical Oncology, Taizhou Cancer Hospital, Taizhou, 317502 Zhejiang China; 10 Department of Anorectal Diseases, Lanxi Hospital of Traditional Chinese Medicine, Jinhua, 321100 Zhejiang China; 11 Division of Clinical Epidemiology and Aging Research, German Cancer Research Center (DKFZ), 69120 Heidelberg, Baden-Württemberg Germany; 12 German Cancer Consortium (DKTK), German Cancer Research Center (DKFZ), 69120 Heidelberg, Baden-Württemberg Germany

**Keywords:** Colorectal cancer (CRC), Screening, Risk-adapted screening, Randomized controlled trial, Cost-effectiveness

## Abstract

**Background:**

Risk-adapted colorectal cancer (CRC) screening has the potential to balance effectiveness with resource demands, yet evidence comparing it with established methods remains limited. This study aims to compare outcomes of risk-adapted CRC screening with colonoscopy and fecal immunochemical test (FIT) strategies.

**Methods:**

We adopted a hybrid methodology combining real-world data from a population-based CRC screening randomized controlled trial (TARGET-C) with projections from a validated Markov-based microsimulation model (MIMIC-CRC). The TARGET-C trial enrolled 19,582 participants aged 50–74 years from 6 centers in China, randomized in a 1:2:2 ratio into 3 groups. After applying the exclusion criteria, the final analysis included 3883 participants in the one-time colonoscopy group, 7793 in the annual FIT group, and 7697 in the risk-adapted screening group. In the latter group, screening allocation was determined by a composite risk score incorporating age, sex, family history of CRC, smoking status, and body mass index, with high-risk participants referred for colonoscopy and low-risk participants for FIT. The primary outcome was detection rates of advanced neoplasm (CRC and advanced adenoma) over 4 rounds. Secondary outcomes included screening participation, colonoscopy demand, and costs from a societal perspective. Long-term effectiveness and cost-effectiveness were modeled over 15 years using MIMIC-CRC.

**Results:**

Across 4 rounds, overall participation rates (attending at least one screening round) were 42.3% (colonoscopy), 99.8% (FIT), and 92.5% (risk-adapted). Detection rates of advanced neoplasms were 2.8%, 2.3%, and 2.6%, respectively, with no significant differences (*P* > 0.05). Colonoscopies needed to detect 1 advanced neoplasm were 15.4, 7.9, and 9.3, respectively. From a societal perspective, the cost for detecting 1 advanced neoplasm was 15,341, 21,754, and 24,300 Chinese Yuan, respectively. Over 15 years, risk-adapted screening reduced incidence by 16.7% and mortality by 21.5% compared with no screening, slightly less effective than colonoscopy (24.6% and 24.8%, respectively). Under observed real-world adherence, colonoscopy was the most cost-effective; under perfect full adherence, risk-adapted screening was the most cost-effective.

**Conclusions:**

In this population-based CRC screening trial, risk-adapted screening, colonoscopy, and FIT demonstrated comparable effectiveness, but differed in participation rates, resource utilization, and cost-effectiveness. Risk-adapted screening could serve as a complementary approach to established strategies, particularly when health resources are limited.

**Trial registration:**

Chinese Clinical Trial Registry (ChiCTR1800015506).

**Supplementary Information:**

The online version contains supplementary material available at 10.1186/s40779-025-00671-7.

## Background

In 2022, more than 1.9 million new colorectal cancer (CRC) cases and 0.9 million deaths occurred worldwide, making CRC the third most commonly diagnosed cancer and the second leading cause of cancer-related mortality [1]. While the benefits of screening for reducing the burden of CRC are widely acknowledged, implementing population-based screening programs remains challenging. Key influencing factors include participation rates, screening yield, healthcare professional engagement, payment mechanisms, and demands on healthcare resources [2–5].

Current CRC screening guidelines recommend a one-size-fits-all approach for average-risk populations, advising colonoscopy or stool-based tests for individuals above a certain age, typically 45 or 50 years [6, 7]. However, individual risk profiles vary significantly. Moreover, available screening modalities differ in terms of test performance, adherence, and resource requirements [8]. Developing precise CRC screening strategies that incorporate factors beyond age to risk-stratify individuals could improve current approaches, optimize resource use, and benefit patients, providers, and healthcare systems [9].

Comparative evaluations against well-established strategies in randomized controlled trials (RCTs) are essential to demonstrate the feasibility and effectiveness of precise risk-adapted CRC screening. Yet, such evidence remains scarce. We conducted a comprehensive multicenter RCT in China, the Comparative Evaluation of Novel Screening Strategies for Colorectal Cancer Screening (TARGET-C), to assess the feasibility, adherence, diagnostic yield, and costs associated with colonoscopy, fecal immunochemical testing (FIT), and a risk-adapted screening approach [10]. Baseline results and 2 rounds of follow-up screenings showed that risk-adapted screening achieved satisfactory participation rates and outcomes comparable to the gold-standard colonoscopy [11, 12]. However, its long-term impact on reducing CRC incidence and mortality requires further exploration.

This study aimed to compare the participation, diagnostic yield, resource demand, and costs of colonoscopy, FIT, and risk-adapted screening over 4 screening rounds in the TARGET-C trial, and to project their long-term effectiveness and cost-effectiveness using a well-validated microsimulation model (MIMIC-CRC) [13] adapted to population characteristics and parameters derived from the TARGET-C trial.

## Methods

### Study design and participants of the TARGET-C trial

The TARGET-C study was an RCT conducted across 6 study centers in 5 provinces of China (Jiangsu, Zhejiang, Anhui, Hunan, and Yunnan) since May 2018. A detailed study protocol has been published previously, with additional details provided in the supplementary materials [10]. Briefly, the study enrolled 19,582 eligible participants aged 50–74 years. Detailed inclusion and exclusion criteria were provided in Additional file 1: Method 1. After obtaining informed consent, participants were randomized in a 1:2:2 ratio into 3 groups: 1) one-time colonoscopy (*n* = 3937); 2) annual FIT (*n* = 7858); or 3) risk-adapted screening (*n* = 7787). In the risk-adapted group, high-risk individuals (based on an established risk score) were referred for colonoscopy, whereas low-risk individuals were referred for FIT. All screening interventions were provided free of charge. Four screening rounds were completed between May 2018 and October 2023, reaching the designated endpoint according to the study protocol. T0–T3 denoted the 4 sequential annual screening rounds, with T0 representing the baseline assessment (year 1) and T1–T3 representing the first (year 2), second (year 3), and third (year 4) follow-up screenings (Additional file 2: Fig. S1). This study presented the final planned results. The study was approved by the Ethics Committee of the National Cancer Center/Cancer Hospital, Chinese Academy of Medical Sciences and Peking Union Medical College (18-013/1615). The trial was registered in the Chinese Clinical Trial Registry (Trial registration: ChiCTN, ChiCTR1800015506; registered April 4, 2018; https://www.chictr.org.cn/showprojEN.html?proj=26005). All participants provided written informed consent.

### Randomization and masking

An individual randomization scheme was generated using R version 3.5.1 software [14]. Allocation was revealed only after successful participant registration. The staff performing randomization were not involved in participant recruitment. Investigators and participants were informed of the allocation after registration, but physicians who performed clinical examinations were blinded to the allocated arms.

### Trial procedures

Eligible participants were recruited from the community by trained staff and registered in an online trial management system that generated a personalized to-do list per trial protocol. Study staff (nurses or public health specialists) contacted participants via phone or instant messaging apps (e.g., WeChat, QQ) to schedule appointments and ensure participation and compliance. Participants visited community health centers to complete a questionnaire, receive trial materials, and have biological samples collected or receive FIT kits, as required. Informed consent was obtained at the community health centers. All clinical examinations were conducted at designated hospitals, and screening interventions were offered free of charge.

Colonoscopy was performed by experienced endoscopists, with abnormal findings sent for pathological examination. If colonoscopy results were negative, participants entered a 10-year surveillance interval. FIT was conducted using a self-administered, qualitative point-of-care test (Pupu Tube, Hangzhou, China; threshold: 8 μg Hb/g feces), with diagnostic characteristics comparable to laboratory-based tests [10]. Participants followed an operation manual to complete FIT at home and uploaded test results via a smartphone app. Uploaded results were verified by study staff to confirm completion and accuracy before diagnostic follow-up. Risk assessment was performed every 2 years (T0 and T2) using the modified Asia-Pacific Colorectal Screening (APCS) Score, which included age (0: 50–54 years; 1: 55–64 years; 2: 65–74 years), sex (0: female; 1: male), family history of CRC among first-degree relatives (0: absent; 1: present), cigarette smoking (0: non-smoker; 1: current or past smoker), and body mass index (BMI, 0: < 23 kg/m^2^; 1: ≥ 23 kg/m^2^) [15]. Participants with a score ≥ 4 were defined as high-risk (referred for colonoscopy), and those with a score < 4 were defined as low-risk (referred for FIT). Colonoscopy and FIT procedures were performed in the same way as previously described.

### Outcome ascertainment

Clinical diagnoses were classified based on the most advanced findings from colonoscopy or histology. CRC was defined as adenocarcinoma of the colon or rectum. Advanced adenoma included adenomas with any of the following: high-grade dysplasia, villous/tubulovillous histology, or size ≥ 10 mm. Advanced serrated adenoma encompassed serrated adenomas (traditional or sessile serrated lesions) ≥ 10 mm or with dysplasia [16]. Both advanced adenomas and serrated lesions were considered advanced precancerous lesions. To ensure consistency, a subset of pathology slides underwent central review by independent pathologists blinded to the original diagnosis.

The primary outcome was the detection rate of advanced colorectal neoplasms (CRC and advanced precancerous lesions). Secondary outcomes included detection of any neoplasm, participation, compliance, and cost. A post hoc power analysis for the detection rate of advanced neoplasm was performed using the *χ*^2^ test.

### Microsimulation analysis for long-term effectiveness and cost-effectiveness

Because evaluating the incidence and mortality of CRC typically requires more than 10 years of observation, we conducted long-term effectiveness and cost-effectiveness analyses using a previously developed microsimulation model (MIMIC-CRC) [13]. A detailed description of the model was provided in Additional file 1: Method 2. Using population characteristics from the TARGET-C study, a simulated cohort of 200,000 individuals (10 times the original sample size) was constructed to represent the target population for long-term screening outcomes. The MIMIC-CRC model was internally calibrated against age-specific CRC incidence and lesion prevalence from the Chinese cancer registry and screening datasets, and externally validated by comparing model-derived hazard ratios for flexible sigmoidoscopy screening with those observed in the UKFSS trial [17]. The cohort was divided into 3 screening groups in a 1:2:2 ratio, each implementing a distinct screening strategy, as defined in the TARGET-C study. Risk status transitions (high-to-low or low-to-high) were permitted at each reassessment, with annual probabilities derived from TARGET-C: 10% for high-to-low and 40% for low-to-high. A 15-year simulation was performed to evaluate the long-term effectiveness and cost-effectiveness of these strategies, focusing on key outcomes such as CRC incidence, mortality, costs, and resource utilization. Resource utilization was assessed by modeling the number of colonoscopies, FIT tests, and diagnostic follow-ups required per strategy. A 15-year simulation horizon was chosen to align with the follow-up duration of major international CRC screening RCTs. This timeframe balances clinical relevance with model stability, as longer horizons introduce greater uncertainty due to demographic and healthcare system changes. Multiple scenario analyses were conducted to assess cost-effectiveness under varying participation rates. Deterministic and probabilistic sensitivity analyses were performed to ensure the robustness of findings.

### Statistical analysis

The study population characteristics were calculated and compared among the 3 arms. The Pearson *χ*^2^ test was used to compare differences in categorical variables. The participation rate was calculated as the number of individuals who completed the designated screening divided by the total number of participants in the corresponding screening group. The overall participation rate for each group was defined as the proportion of individuals who attended at least 1 round of screening during the 4 screening rounds. The participation rates and their corresponding 95% confidence intervals (CIs) were estimated using the Wilson method. We used multinomial non-conditional logistic regression models to calculate odds ratios (*ORs*) and 95% CIs for colorectal neoplasm detection rates, adjusting for age, sex, and study center. The main results were reported according to the intention-to-treat (ITT) principle (i.e., participants were analyzed in their original allocation arm, regardless of screening status). Per-protocol (PP) analyses (defined as participants undergoing screening per protocol) were also reported. Subgroup analyses were conducted by sex and anatomical location of neoplasms. Costs were estimated from both societal and government perspectives. Detailed methods and aggregated cost parameters were provided in the supplement. The cost per neoplasm detected was used as a key indicator across screening arms and rounds. All costs were reported in Chinese Yuan (CNY) and US dollars (1 USD = 6.8996 CNY, based on the 2020 annual average exchange rate). In the modeling analyses, we estimated year-by-year health outcomes, costs, and resource utilization for the TARGET-C population over 15 years. Primary outcomes were CRC incidence, CRC-related mortality, costs, and quality-adjusted life-years (QALYs) of the different screening strategies from a societal perspective. To compare the cumulative incidence and mortality of CRC between groups, the percentage change was computed as the difference between the screening and no-screening groups divided by the number of cases in the no-screening group. We also calculated incremental cost-effectiveness ratios (ICERs) by dividing incremental costs by incremental QALYs gained for each strategy, discounted at 5%, following China’s healthcare economic guidelines. Strategies were ranked by ICERs relative to the next-best alternative, and the cost-effectiveness frontier was identified at different willingness-to-pay (WTP) thresholds. Strategies with ICERs of 0.5- and 2-times China’s gross domestic product per capita (CNY 35,446 and 141,784) per QALY gained were classified as strongly and weakly cost-effective, respectively. The median of 1000 iterations of the best parameter set was used to represent the point estimates, with 95% uncertainty intervals (UI) to quantify uncertainty. Deterministic and probabilistic sensitivity analyses were conducted to assess the sensitivity of the results to changes in model input parameters. All analyses were performed using R version 4.4.0 software [14]. All tests were two-sided, with statistical significance set at a *P*-value < 0.05.

## Results

### Study population characteristics

**Fig. 1:**
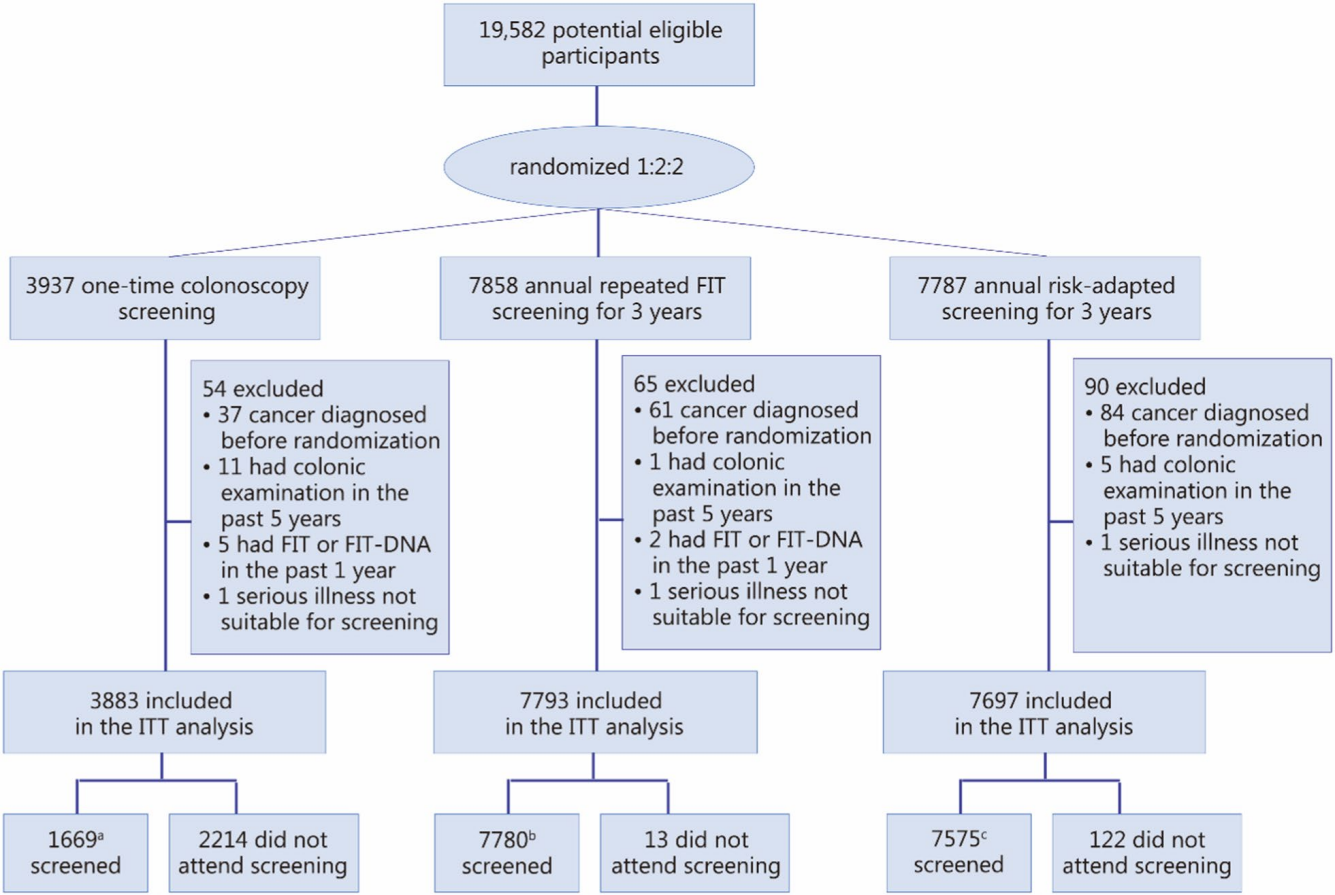
Flow diagram of the study participants. ^a^1644 had colonoscopy screening per protocol at baseline, 2 had colonoscopy, and 8 had FIT at the third round of screening, 1 had colonoscopy and 14 had FIT at the fourth round of screening. ^b^7775 had attended at least 1 round of screening per protocol, and 5 examinations without having FIT or with a negative FIT result. ^c^7122 had attended at least 1 round of screening per protocol, 103 underwent colonoscopy screening among low-risk individuals without having FIT or with negative FIT results, and 350 had FIT screening among high-risk individuals. FIT fecal immunochemical test, ITT intention-to-treat

A total of 19,582 participants were recruited, of whom 209 were excluded for not meeting the inclusion criteria. The final analysis included 19,373 participants, with 3883 in the colonoscopy group, 7793 in the FIT group, and 7697 in the risk-adapted screening group (Fig. 1). Characteristics of the study population are shown in Additional file 2: Table S1. Among all participants, 8022 (41.7%) were men, with a mean age of 60.5 years. The distribution of basic characteristics was generally comparable across the 3 study arms, except for a slightly higher proportion of participants with a family history of CRC in the risk-adapted group.

### Participation and compliance

Additional file 2: Table S2 and Fig. S1 illustrate the participation across the 3 arms from the T0 to T3 phases. The overall participation declined for both the FIT arm and the risk-adapted screening arm. In the FIT arm, participation at baseline was 94.0% (95% CI 93.5–94.5), which gradually decreased in subsequent rounds, although it remained relatively high (> 80%). In the risk-adapted screening arm, baseline participation was 85.2% (95% CI 84.4–86.0), declining to 63.4% (95% CI 62.3–64.5) by the 4th round. Additional file 2: Table S3 illustrates the participation of the risk-adapted screening arm, categorized by risk profile, from the T0 to T3 phases. In the risk-adapted arm, FIT participation rates among low-risk individuals were similarly high, ranging from 94.0% (95% CI 93.4–94.6) at baseline to 78.8% (95% CI 77.7–79.9) in the 4th round. However, among high-risk individuals, the colonoscopy participation rate was 49.0% (95% CI 46.4–51.6) at baseline; in the following 3 rounds, this rate decreased to 6.4% (95% CI 4.8–8.5), 10.5% (95% CI 8.6–12.7), and 3.7% (95% CI 2.7–5.0). Additional file 2: Table S4 illustrates the cumulative participation across the 3 arms from the T0 to T3 phases. The overall participation rates (attending at least 1 screening round) were 42.3% (1644/3883, 95% CI 40.8–43.9) in the colonoscopy arm, 99.8% (7775/7793, 95% CI 99.6–99.9) in the FIT arm, and 92.5% (7122/7697, 95% CI 91.9–93.1) in the risk-adapted arm. Regarding colonoscopy compliance among FIT-positive participants across the 4 screening rounds, the compliance rates in the FIT arm were 76.3% (817/1071), 75.7% (258/341), 71.7% (243/339), and 66.9% (81/121) for rounds 1 through 4, respectively, yielding an overall compliance rate of 74.7% (1399/1872). In the risk-adapted screening arm, the corresponding compliance rates among low-risk participants were 76.9% (601/782), 74.6% (182/244), 60.1% (98/163), and 63.5% (66/104) across the same rounds, with an overall compliance rate of 73.2% (947/1293) (Additional file 2: Fig. S1).

### Detection rate for advanced neoplasm

**Fig. 2:**
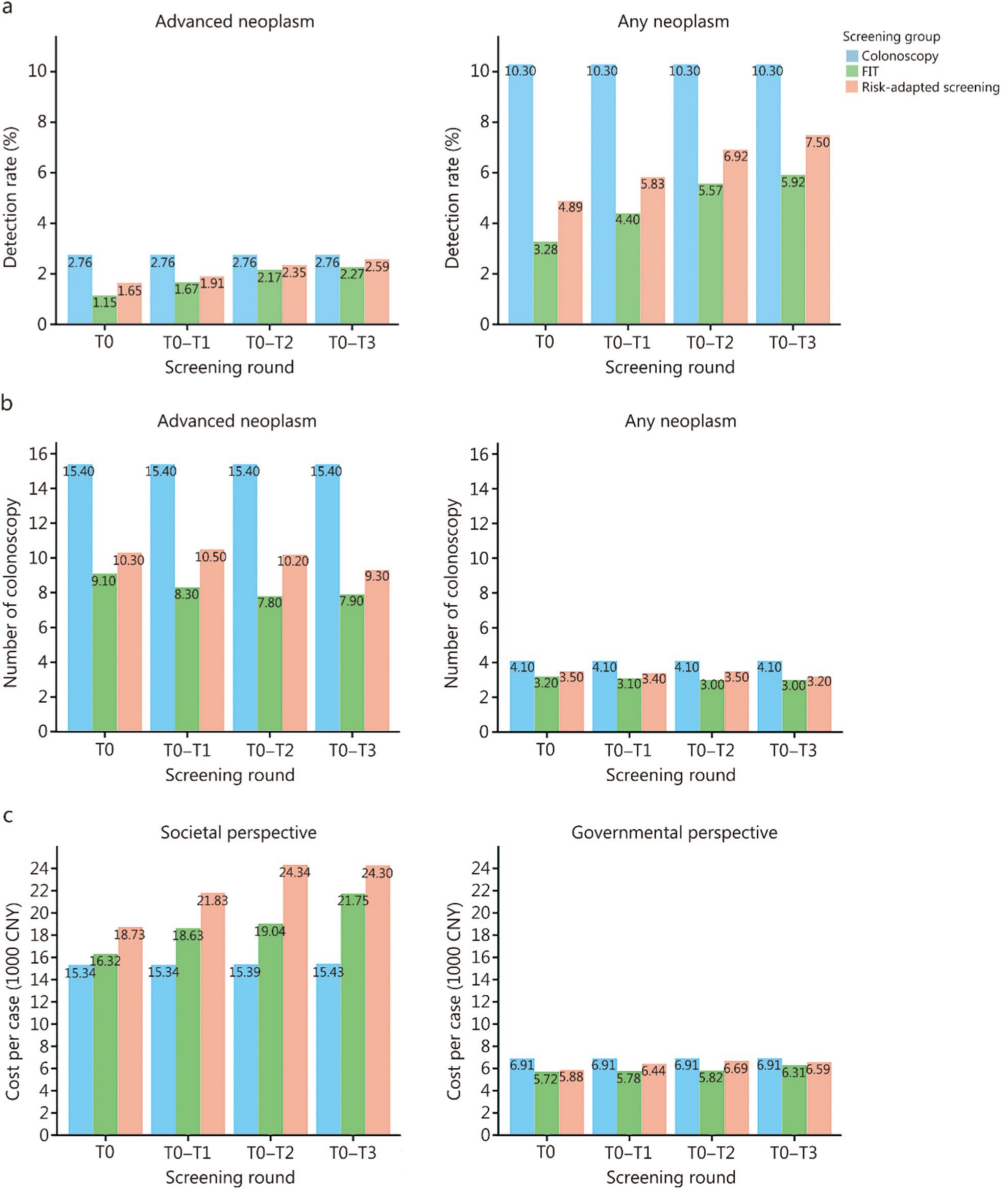
Comparison of screening yield and cost-effectiveness among different arms over 4 rounds of screening. **a** Detection rate for detecting advanced neoplasm or any neoplasm (ITT analysis). **b** Number of colonoscopies needed to be performed to detect 1 advanced neoplasm or any neoplasm. **c** Cost (in CNY 1000) for detecting 1 advanced neoplasm from the societal perspective and government perspective. CNY Chinese Yuan, FIT fecal immunochemical test, ITT intention-to-treat

Screening yield results from the ITT analysis are presented in Table 1, Fig. 2a, and Additional file 2: Table S5. The cumulative detection rates of advanced neoplasms over the 4 screening rounds were 2.8% (95% CI 2.3–3.3), 2.3% (95% CI 2.0–2.6), and 2.6% (95% CI 2.3–3.0) in the colonoscopy, FIT, and risk-adapted screening arms, respectively (Fig. 2a and Additional file 2: Table S5). After adjusting for age, sex, and study center, the *ORs* for the cumulative detection rate of advanced neoplasms were 1.21 (95% CI 0.95–1.55, *P* = 0.124) for colonoscopy vs. FIT, 1.06 (95% CI 0.83–1.34, *P* = 0.658) for colonoscopy vs. risk-adapted screening, and 1.15 (95% CI 0.93–1.41, *P* = 0.197) for risk-adapted screening vs. FIT (Table 1 and Additional file 2: Table S5). We observed that the risk-adapted screening arm had a slightly higher detection rate of advanced neoplasms among men compared to the FIT arm (*OR* = 1.30, 95% CI 1.01–1.67, *P* = 0.046), while the colonoscopy arm showed a higher detection rate among women compared to the risk-adapted screening arm (*OR* = 1.52, 95% CI 1.00–2.28, *P* = 0.046) (Table 1 and Additional file 2: Table S5). No statistically significant differences were found in the detection of advanced neoplasms in the proximal or distal colon/rectum across the 3 arms. A post hoc power analysis demonstrated 82.4% power to detect the observed differences in detection rates (Cohen’s *w* = 0.043), with a *χ*^2^ statistic of 4.76 (*df* = 2, *P* = 0.093). The results of detection rates for any colorectal neoplasm are shown in Additional file 2: Table S6.

**Table 1:** Numbers and proportions of participants with detected colorectal neoplasms after 4 rounds of screening (ITT analysis)

Colorectal neoplasm	Colonoscopy [*n* = 3883,* n* (%)]	FIT [*n* = 7793, *n* (%)]	Risk-adapted screening [*n* = 7697, *n* (%)]	Colonoscopy vs. FIT		Colonoscopy vs. Risk-adapted screening		Risk-adapted screening vs*.* FIT	
				*OR* (95% CI) ^a^	*P*-value	*OR* (95% CI)^a^	*P*-value	*OR* (95% CI)^a^	*P*-value
Overall									
Colorectal cancer	10 (0.3)	10 (0.1)	15 (0.2)	2.03 (0.83–4.96)	0.113	1.32 (0.57–2.91)	0.497	1.53 (0.70–3.53)	0.295
Advanced precancerous lesion	97 (2.5)	167 (2.1)	184 (2.4)	1.16 (0.90–1.50)	0.252	1.03 (0.80–1.33)	0.802	1.12 (0.90–1.39)	0.295
Non-advanced adenoma	293 (7.6)	284 (3.6)	378 (4.9)	2.21 (1.86–2.62)	< 0.001	1.61 (1.37–1.89)	< 0.001	1.39 (1.18–1.63)	< 0.001
Advanced neoplasm	107 (2.8)	177 (2.3)	199 (2.6)	1.21 (0.95–1.55)	0.124	1.06 (0.83–1.34)	0.658	1.15 (0.93–1.41)	0.197
Any neoplasm	400 (10.3)	461 (5.9)	577 (7.5)	1.89 (1.63–2.18)	< 0.001	1.45 (1.26–1.67)	< 0.001	1.32 (1.15–1.50)	< 0.001
Advanced neoplasm									
Proximal colon ^b^	61 (1.6)	95 (1.2)	96 (1.2)	1.28 (0.92–1.77)	0.133	1.26 (0.90–1.74)	0.170	1.02 (0.77–1.36)	0.883
Distal colon/rectum ^b^	73 (1.9)	125 (1.6)	154 (2.0)	1.16 (0.86–1.56)	0.324	0.92 (0.69–1.22)	0.562	1.26 (0.99–1.61)	0.060
Men	66 (4.1)	115 (3.5)	143 (4.5)	1.14 (0.83–1.56)	0.409	0.88 (0.65–1.19)	0.409	1.30 (1.01–1.67)	0.046
Women	41 (1.8)	62 (1.4)	56 (1.2)	1.35 (0.90–2.01)	0.144	1.52 (1.00–2.28)	0.046	0.89 (0.62–1.28)	0.531
Any neoplasm									
Proximal colon ^b^	199 (5.1)	236 (3.0)	276 (3.6)	1.75 (1.44–2.13)	< 0.001	1.46 (1.21–1.77)	< 0.001	1.20 (1.00–1.44)	0.047
Distal colon/rectum ^b^	249 (6.4)	296 (3.8)	388 (5.0)	1.77 (1.48–2.11)	< 0.001	1.30 (1.09–1.53)	0.003	1.37 (1.17–1.60)	< 0.001
Men	232 (14.4)	280 (8.5)	405 (12.8)	1.82 (1.50–2.20)	< 0.001	1.13 (0.94–1.36)	0.176	1.60 (1.36–1.89)	< 0.001
Women	168 (7.4)	181 (4.0)	172 (3.8)	1.99 (1.59–2.48)	< 0.001	2.15 (1.71–2.69)	< 0.001	0.93 (0.75–1.16)	0.540

^a^Odds ratios and 95% confidence intervals were adjusted for age, sex (not for subgroup analysis of men and women), and participating center in the logistic regression models

^b^The sum may exceed the total number because of the possibility of findings in both the proximal and distal colon/rectum

*CI* confidence interval, *FIT* fecal immunochemical test, *ITT* intention-to-treat, *OR* odds ratio

In the PP analyses (Additional file 2: Table S7), detection rates of advanced neoplasms in the colonoscopy, FIT, and risk-adapted screening arms were 6.5%, 2.3%, and 2.7%, respectively, with an adjusted *OR*_colonoscopy vs. FIT_ of 2.42 (95% CI 1.87–3.11, *P* < 0.001), an adjusted *OR*_colonoscopy vs. risk-adapted screening_ of 1.90 (95% CI 1.48–2.44, *P* < 0.001), and an adjusted *OR*_risk-adapted screening vs. FIT_ of 1.32 (95% CI 1.07–1.63, *P* = 0.011).

### Resource demand and costing outcomes

Detailed resource utilization and cost analysis results are shown in Fig. 2b, c, and Additional file 2: Tables S8–S10. In the colonoscopy arm, 15.4 colonoscopies were needed to detect 1 advanced neoplasm. In the FIT arm, the number of colonoscopies needed to detect 1 advanced neoplasm was 9.1, 8.3, 7.8, and 7.9 at T0, T0–T1, T0–T2, and T0–T3, respectively. In the risk-adapted screening arm, the corresponding values were 10.3, 10.5, 10.2, and 9.3 (Fig. 2b and Additional file 2: Table S8). From a societal perspective (Fig. 2c and Additional file 2: Table S9), the costs of detecting 1 advanced neoplasm were CNY 15,341 ($ 2223) for colonoscopy, CNY 21,754 ($ 3153) for FIT, and CNY 24,300 ($ 3522) for risk-adapted screening. From a government perspective (Fig. 2c and Additional file 2: Table S10), the costs for detecting 1 advanced neoplasm were comparable between the 3 strategies, with CNY 6914 ($ 1002) for colonoscopy, CNY 6313 ($ 915) for FIT, and CNY 6589 ($ 955) for risk-adapted screening.

### Effectiveness on CRC incidence and mortality

**Fig. 3:**
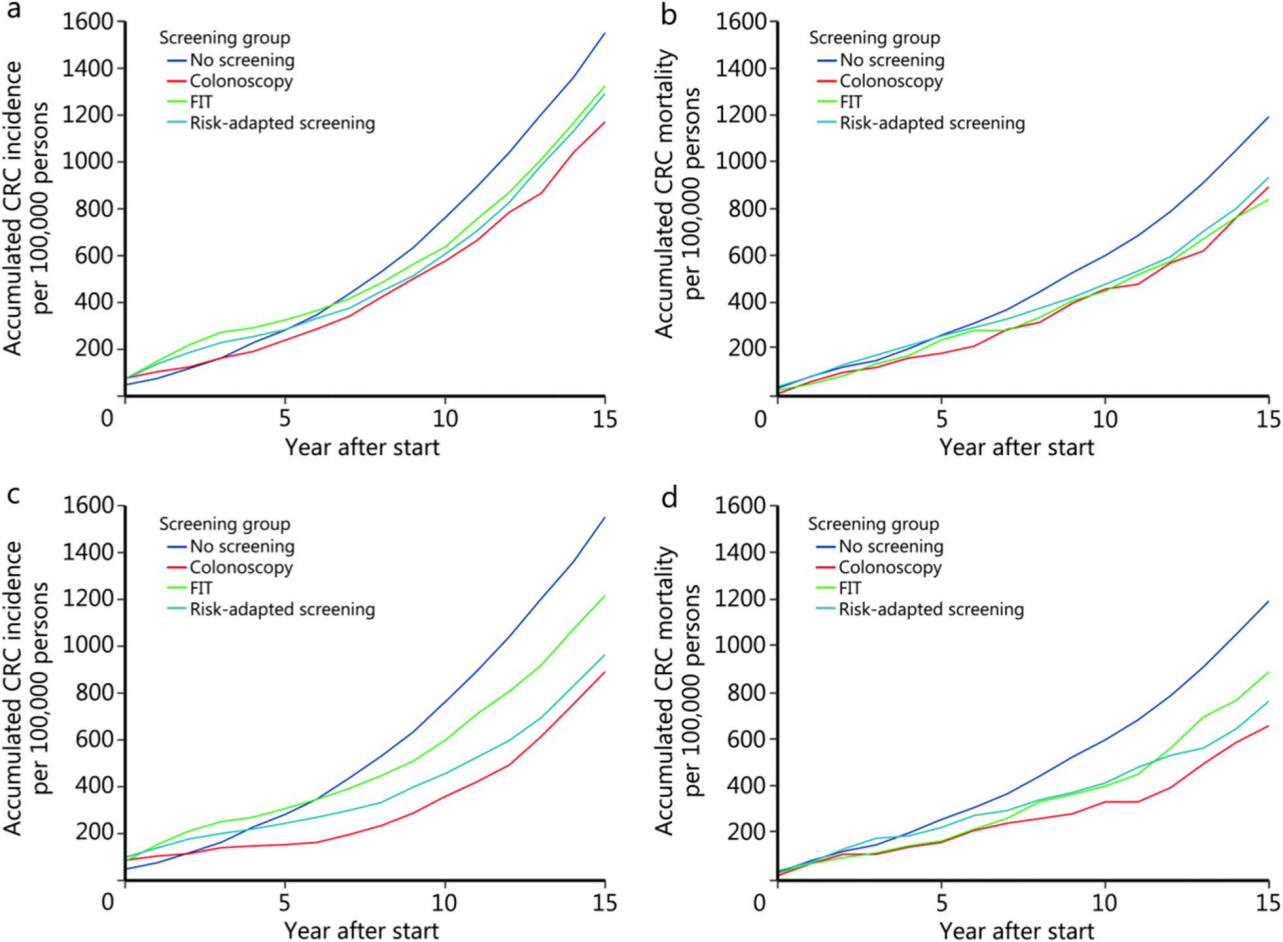
Long-term predicted colorectal cancer incidence and mortality of the 3 different screening arms and no screening. **a** Incidence under the status quo. **b** Mortality under the status quo. **c** Incidence under 100% adherence scenario. **d** Mortality under 100% adherence scenario. CRC colorectal cancer, FIT fecal immunochemical test

Figure 3 and Additional file 2: Table S11 present the projected CRC incidence and mortality across the 3 screening arms and the no-screening scenario over 15 years. Over the 15 years, all screening strategies were associated with substantial reductions in both CRC incidence and mortality compared with no screening. Under the observed real-world adherence, the cumulative CRC incidence per 100,000 persons over 15 years was 1171, 1325, and 1293 for the colonoscopy, FIT, and risk-adapted screening arms, respectively, and 1552 for the no-screening group, corresponding to relative reductions of 24.6%, 14.6%, and 16.7%. The cumulative CRC mortality per 100,000 persons over the same period was 224, 210, and 234 for the colonoscopy, FIT, and risk-adapted screening arms, respectively, and 298 for the no-screening group, corresponding to reductions of 24.8%, 29.5%, and 21.5% relative to no screening. Therefore, in the TARGET-C trial (status quo), colonoscopy screening outperformed both FIT and risk-adapted screening. In addition, under the ideal scenario of 100% adherence, all strategies showed further improvements, with colonoscopy remaining the most effective in reducing CRC incidence and mortality. Compared with no screening, the relative reductions in CRC incidence and mortality were 41.0% and 45.3% for colonoscopy, 16.9% and 25.5% for FIT, and 37.9% and 35.9% for risk-adapted screening, respectively.

### Cost-effectiveness analysis

Table 2 presents the cost-effectiveness analyses of the screening strategies over 15 years. All screening strategies were more cost-effective than no screening, using a WTP threshold of CNY 141,784 per QALY saved. Under the current scenario, colonoscopy screening yielded the highest discounted QALY per person at 9.631 (95% UI 9.618–9.645), with an ICER of 9060 CNY/QALY compared with no screening after 15 years. Within 15 years, risk-adapted screening and FIT screening were both dominated by colonoscopy.

**Table 2:** Cost-effectiveness analyses of colonoscopy group, fecal immunochemical test group and the risk-adapted screening group in modeling analysis over 15 years

Scenario	Year after start	Screening strategy^a^	Discounted QALY per person (95% UI)^†^	Discounted cost per person (CNY, 95% UI)^†^	Colonoscopy used per 100,000 persons	ICER compared to no screening (median, CNY/QALY)	ICER compared to colonoscopy screening (median, CNY/QALY)
Status quo	5	No screening	4.857 (4.855–4.860)	161 (152–166)	289	Ref	–
		Colonoscopy screening	4.864 (4.863–4.866)	530 (502–547)	42,659	52,307	Ref
		FIT screening	4.860 (4.858–4.867)	633 (599–653)	22,074	157,590	Dominated
		Risk-adapted screening	4.860 (4.859–4.862)	788 (746–813)	41,836	241,594	Dominated
	10	No screening	7.677 (7.671–7.683)	344 (326–355)	747	Ref	–
		Colonoscopy screening	7.696 (7.689–7.702)	665 (630–686)	42,997	16,631	Ref
		FIT screening	7.685 (7.677–7.692)	757 (717–781)	22,386	51,812	Dominated
		Risk-adapted screening	7.685 (7.679–7.690)	917 (868–946)	42,159	69,102	Dominated
	15	No screening	9.604 (9.591–9.617)	603 (571–622)	1568	Ref	–
		Colonoscopy screening	9.631 (9.618–9.645)	852 (807–879)	43,592	9060	Ref
		FIT screening	9.621 (9.610–9.632)	975 (923–1006)	23,075	21,882	Dominated
		Risk-adapted screening	9.618 (9.606–9.631)	1132 (1072–1168)	42,846	37,524	Dominated
100% adherence	5	No screening	4.857 (4.829–4.886)	161 (152–166)	289	Ref	–
		Colonoscopy screening	4.863 (4.861–4.865)	1041 (986–1074)	100,123	145,224	Ref
		FIT screening	4.860 (4.857–4.865)	680 (644–702)	24,713	173,269	–
		Risk-adapted screening	4.866 (4.863–4.871)	1136 (1076–1172)	83,982	113,460	37,483
	10	No screening	7.677 (7.671–7.683)	344 (326–355)	747	Ref	–
		Colonoscopy screening	7.691 (7.686–7.698)	1119 (1060–1155)	100,324	54,157	Ref
		FIT screening	7.684 (7.675–7.693)	806 (763–832)	25,030	66,265	–
		Risk-adapted screening	7.696 (7.689–7.705)	1219 (1154–1258)	84,194	45,359	20,076
	15	No screening	9.604 (9.591–9.617)	603 (571–622)	1568	Ref	–
		Colonoscopy screening	9.623 (9.611–9.637)	1285 (1217–1326)	100,856	35,011	Ref
		FIT screening	9.620 (9.609–9.632)	1011 (957–1043)	25,684	25,500	–
		Risk-adapted screening	9.634 (9.620–9.646)	1378 (1305–1422)	84,703	25,749	8759

^a^The participation rate of status quo in the colonoscopy screening group is 42.34%; the participation rate of status quo in the FIT screening group is 94.62%; the participation rate of status quo in the risk-adapted screening group is 49.00% for the high-risk group and 94.60% for the low-risk group. ^†^The discount rate is 5% per year

*CNY* Chinese Yuan*, FIT* fecal immunochemical test, *ICER* incremental cost-effectiveness ratio*, QALY* quality-adjusted life year, *UI* uncertainty interval

To explore the impact of adherence on cost-effectiveness, we varied the probabilities of colonoscopy and FIT uptake (Additional file 2: Fig. S2a, b). FIT was the most cost-effective strategy in most scenarios when colonoscopy adherence fell to 20%. As colonoscopy adherence increased to 80%, colonoscopy screening became the most cost-effective approach in a greater number of scenarios. However, under the assumption of 100% adherence for both colonoscopy and FIT, the risk-adapted screening strategy was identified as the most cost-effective. The cost-effectiveness plane plot (Additional file 2: Fig. S2c), which illustrates this specific 100% adherence scenario, confirms the cost-effectiveness of the risk-adapted strategy under this condition.

### Sensitivity analysis

In the sensitivity analysis, one-way analyses revealed that the ICERs of the screening strategies were sensitive to changes in the costs of FIT and colonoscopy, as well as transition rates across different disease states (Additional file 2: Fig. S3). Probabilistic sensitivity analyses and cost-effectiveness acceptability curves showed that colonoscopy screening had the highest probability of being cost-effective at the WTP threshold of 141,784 CNY/QALY (Additional file 2: Fig. S4).

## Discussion

This study provides a comparative evaluation of participation, screening yield, and the long-term impact of colonoscopy, FIT, and risk-adapted screening approaches on CRC incidence and mortality. Across 4 screening rounds, the risk-adapted screening approach demonstrated high participation rates, with a screening yield for advanced neoplasms comparable to colonoscopy but with a reduced demand for endoscopy resources. Microsimulation modeling demonstrated that colonoscopy was the most effective screening modality, achieving the largest reductions in incidence and mortality [18]. However, its low participation rate limits population-level impact, and cost-effectiveness highly depends on adherence to colonoscopy or FIT.

Repeated screening is critical for CRC prevention. Although the detection rates of CRC and advanced precancerous lesions were slightly lower in the risk-adapted and FIT arms during the first round of screening compared with the colonoscopy arm, additional neoplasms were identified in subsequent rounds. After 4 rounds of screening, no statistically significant differences were observed in advanced neoplasm detection among the arms, consistent with findings from previous large-scale studies [19–21]. Secondary outcomes revealed statistically significant differences in neoplasm detection rates across screening strategies. Colonoscopy demonstrated superior detection of non-advanced adenomas compared with FIT and risk-adapted screening, likely reflecting its higher sensitivity for smaller lesions. However, this advantage did not translate to statistically significant differences in advanced neoplasm detection rates, suggesting that noninvasive strategies may achieve comparable performance for clinically significant lesions while reducing colonoscopy demand. It is worth noting that the marginal benefits of detecting additional cases diminish over time. The dynamic risk-adapted approach balances precision and resource use. Biennial reassessment allows timely identification of emerging risks, while limiting colonoscopies to initial high-risk cases prevents overuse. The modeled risk transitions (10% high-to-low and 40% low-to-high annually) align with real-world longitudinal data, supporting the strategy’s adaptability without compromising effectiveness. Consequently, the question of whether to extend screening intervals for participants with multiple rounds of negative findings warrants further investigation, which is beyond the scope of this trial.

Determining the long-term impact of different CRC screening strategies is crucial for translating findings into population-based programs; however, such evaluations typically require rigorously designed RCTs with over 10 years of follow-up. Current RCTs evidence is limited to fecal occult blood test [16, 22], flexible sigmoidoscopy [17, 23–25], and colonoscopy [26], with the TARGET-C trial being the only RCTs assessing risk-adapted screening [9]. To provide timely evidence, we used the validated MIMIC-CRC microsimulation model [13] to predict the long-term impact on CRC burden. Comparable models, such as MISCAN, CRC-SPIN, and SIM-CRC, have been well developed and widely used to inform screening guidelines [18, 27]. Similarly, the MIMIC-CRC model, calibrated with parameters from the trial, provides credible predictions. However, validation using other microsimulation models tailored to different populations would further improve generalizability.

Our findings indicate that risk-adapted screening can reduce CRC incidence and mortality to levels comparable with colonoscopy, while also lowering demand for colonoscopy resources. Although colonoscopy and FIT screening are cost-effective in specific scenarios, particularly when adherence rates are high, risk-adapted screening remains the most cost-effective strategy across adherence combinations. In the long term, risk-adapted screening offers substantial economic benefits by reducing CRC incidence and late-stage diagnoses, thereby reducing the high costs of cancer treatment. While the initial implementation costs of risk-adapted screening may be higher due to the complexity of its protocol, these costs are offset by reductions in cancer-related healthcare expenditures and improvements in QALYs.

An effective risk-prediction model is the core of risk-adapted screening. Most well-established risk prediction models incorporate personal and lifestyle risk factors, such as age, sex, family history of CRC among first-degree relatives, BMI, and smoking [9]. Although the model derivation method differed among these models, their predictive performance was comparable for different populations, with the areas under the receiver operating characteristic (ROC) curve ranging from 0.61 to 0.70 in a meta-analysis [28]. The APCS score used in the current study utilizes common personal and lifestyle risk factors and has been validated in previous research [15]. Its generalizability is most likely assured when applied to other populations. However, the APCS risk score’s reliance on age and sex disproportionately categorizes women as low-risk, potentially under-referring them for colonoscopy. While this reflects lower CRC incidence in younger women, the subgroup analysis revealed that colonoscopy detected significantly more neoplasms in women than risk-adapted screening. This raises ethical concerns, as sex-based under-referral could inadvertently delay diagnosis in women with atypical risk profiles. Ensuring equity in screening recommendations requires further validation of risk scores across sex-specific subgroups. Future refinements may consider sex-specific thresholds or incorporate additional risk factors to reduce the risk of systematic under screening. Emerging genomic and epigenomic markers are increasingly being explored for CRC screening and early detection. Polygenic risk scores combined with lifestyle and clinical factors may support more individualized risk communication [29, 30], while next-generation molecular stool assays using multitarget DNA or RNA markers have also demonstrated encouraging performance in large prospective, colonoscopy-controlled studies [31, 32]. Despite their potential to complement or refine current noninvasive tests, critical questions remain regarding their cost-effectiveness, implementation feasibility, and equitable access. These aspects require careful evaluation in diverse healthcare settings before integration into routine population-based screening programs.

Participation is essential for determining the overall screening yield, yet improving participation and compliance is a major challenge in population-based CRC screening. The results in this study demonstrated that the overall compliance rate for colonoscopy was low, consistent with findings from previous population-based studies [26, 33]. A critical limitation of risk-adapted screening is the low colonoscopy participation rate among high-risk individuals, which may diminish the theoretical benefits of this strategy. Future implementations should incorporate evidence-based adherence-enhancing measures, such as reminder systems, patient navigation, personalized communication, or financial incentives. These strategies, which have proven effective in improving participation in other cancer screening programs, could further strengthen the effectiveness of risk-adapted screening in real-world practice. Participation is even lower in a real-world screening setting [34]. We demonstrated that noninvasive testing and risk assessment significantly enhance overall participation, particularly after multiple rounds of screening. The integration of novel blood-based biomarkers into risk-adapted screening warrants further investigation to optimize effectiveness.

Our study has some limitations. First, participation in colonoscopy and compliance with diagnostic follow-up among FIT-positive individuals were suboptimal, with noncompliance primarily observed in older participants with comorbidities, reflecting real-world barriers to colonoscopy acceptance across diverse populations. Second, in the risk-adapted screening arm, high-risk individuals who declined colonoscopy were not offered alternative screening modalities per protocol, to avoid bias when assessing repeated screening yields. Third, the absence of re-invitations for colonoscopy in the one-time screening arm prevented direct comparisons of longitudinal participation dynamics between strategies. While the risk-adapted arm allowed repeated colonoscopy referrals for high-risk non-responders, the colonoscopy arm’s protocol restricted screening to T0 only. Fourth, while the microsimulation model provided robust projections, its accuracy depended on the quality of input data for risk stratification and disease progression. It did not account for potential changes in population characteristics or healthcare policies over time, which could affect future applicability. Fifth, the exclusion of serrated polyps from the MIMIC-CRC model might have influenced the results. Finally, because study parameters and populations were derived from China, the generalizability of the findings to other settings or populations might be limited.

## Conclusions

In summary, in this population-based CRC screening trial, risk-adapted screening, colonoscopy, and FIT showed comparable effectiveness but differed in participation, resource use, and cost-effectiveness. By prioritizing colonoscopy for high-risk individuals and FIT for low-risk individuals, this approach complements existing screening modalities, particularly in resource-limited settings.

## Declaration

## Supplementary Information

**Additional file 1.** Method 1 and Method 2.

**Additional file 2. Table S1** Baseline study population characteristics among 3 screening arms [*n* (%)]. **Table S2** Participation across the 3 screening arms from the T0 to T3 phases. **Table S3** Participation of the risk-adapted screening arm from the T0 to T3 phases. **Table S4** Cumulative participation across the 3 screening arms from the T0 to T3 phases. **Table S5** Detection for advanced colorectal neoplasm among invited individuals in the colonoscopy arm, fecal immunochemical test arm, and the risk-adapted screening arm for the 4 screening rounds (ITT analysis). **Table S6** Detection for any colorectal neoplasm among invited individuals in the colonoscopy arm, fecal immunochemical test arm, and the risk-adapted screening arm for the 4 screening rounds (ITT analysis). **Table S7** Detected colorectal neoplasm of the included participants in the colonoscopy arm, fecal immunochemical test arm, and the risk-adapted screening arm for the cumulative 4 rounds of screening (PP analysis). **Table S8** Number of colonoscopies needed to be performed to detect 1 neoplasm (ITT analysis) [number of colonoscopies (95% CI)]. **Table S9** The costs per case detected, by trial arm and by screening round, from a societal perspective. **Table S10** The costs per case detected, by trial arm and by screening round, from a government perspective. **Table S11** Projected 5-, 10-, and 15-year incidence and mortality from the modeling analysis. **Fig. S1** Detailed scheme of the study participants in the colonoscopy arm, fecal immunochemical test arm (FIT), and the risk-adapted screening arm from the T0 to T3 phases. **Fig. S2** Cost-effectiveness analyses between screening arms under different scenarios with varying colonoscopy and fecal immunochemical test (FIT) adherence. **Fig. S3** Result of the determined sensitivity analysis of colonoscopy screening compared to fecal immunochemical test screening (a) and risk-adapted screening (b). **Fig. S4** Result of probabilistic sensitivity analysis.

## Data Availability

Access to the deidentified participant data and code for the statistical analysis will be granted based on reasonable requests to the corresponding authors (chenhongda@pumch.cn; daimin2002@hotmail.com).

## Abbreviations

APCS: Asia-Pacific Colorectal Screening
CI: Confidence interval
CNY: Chinese Yuan
CRC: Colorectal cancer
FIT: Fecal immunochemical testing
ICER: Incremental cost-effectiveness ratio
ITT: Intention-to-treat
OR: Odds ratio
PP: Per-protocol
QALY: Quality-adjusted life-year
RCT: Randomized controlled trial
UI: Uncertainty interval
