# Supplementary material for: Effectiveness and cost-effectiveness of risk-adapted colorectal cancer screening: a randomized controlled trial and modeling analysis
Source: Mil Med Res. 2025 Nov 24;12:82. doi: 10.1186/s40779-025-00671-7 (PMC12642085; doi:10.1186/s40779-025-00671-7)
Supplement: Supplementary file 1 — Additional file 1. Method 1 and Method 2. [file 40779_2025_671_MOESM1_ESM.pdf]

Method 1

Inclusion and exclusion criteria

Inclusion criteria

1) Individuals aged 50 – 74 years living in the study region.
2) Able to sign informed consent.

Exclusion criteria

1) Prior history of colorectal cancer (CRC).
2) Prior history of colonic resection.
3) Receipt of any kind of cancer-related therapy (except for non-melanoma skin cancer).
4) Prior colonic examination, including colonoscopy, flexible sigmoidoscopy, computed tomography (CT) colonography, and barium enema within 5 years.
5) Prior history of fecal occult blood test and fecal DNA test within 1 year.
6) Symptoms of lower gastrointestinal tract disease warranting colonoscopic evaluation, including: i) more than one episode of rectal bleeding within the past 6 months; ii) documented iron deficiency anemia, and iii) significant documented unintentional weight loss (> 10% of baseline weight) over 6 months.
7) Significant comorbidity that would preclude benefit from screening or pose a significant risk to the performance of colonoscopy (e.g., severe lung disease, end-stage renal disease, end-stage liver disease, severe heart failure, or recent diagnosis of cancer, except for non-melanoma skin cancer.

Detailed trial procedures regarding colonoscopy, fecal immunochemical test, and risk-adapted screening

Colonoscopy

All colonoscopies were performed by experienced endoscopists following standard procedures. Abnormal findings during colonoscopy were carefully checked and sent for pathology examination under standard clinical procedures. Any findings during a colonoscopy were required to be documented photographically. Clinical information, such as the examination duration, sedation status, completeness of colonoscopy, bowel preparation status, complications, polyp features, description of other abnormal findings, as well as pathology diagnosis, was collected and documented in the web-based data management system.

To ensure the consistency of pathology diagnosis among different hospitals, we conducted a central review of the pathology sections for all CRCs, all advanced adenomas, and a random selection of 10% of the non-advanced adenomas by an experienced gastrointestinal pathologist from the National Cancer Center of China. Any inconsistent diagnosis was resolved by consensus of at least 2 central pathologists.

Fecal immunochemical test (FIT)

A self-administered qualitative FIT for hemoglobin (Pupu Tube, New Horizon Health Technology, China) was used. In our previous validation, the sensitivities of the Pupu-FIT for detecting CRC and advanced adenoma were 83.3% and 33.9%, respectively, at a specificity of 90.2%, and in a head-to-head comparison with a quantitative FIT (OC-sensor, Eiken, Japan), both tests had comparable test performance when adjusting to yield similar specificity [1]. Study staff members distributed the FIT to the participants when they visited the community health service center and instructed the participants on the operating procedures. The participants were recommended to undertake FIT at home or at the community health service center if possible. The operation manual is provided in Section 3. Briefly, the FIT enables visual interpretation of the test results as positive or negative by eye if the fecal hemoglobin (Hb) concentration exceeded the threshold specified by the manufacturer (100 ng Hb/ml, equivalent to 8 μg Hb/g feces). The participants were instructed to submit the test results to the study website via smartphone app, along with a picture of the test window of the FIT, or were interviewed by the study staff. Participants with confirmed positive FIT results were scheduled for a subsequent diagnostic colonoscopy, which followed the same procedure described previously.

Risk assessment

We used the modified Asia-Pacific Colorectal Screening Score (APCS) for risk stratification (**Table S1**), which

comprised 5 CRC-related risk factors, including age, sex, family history of CRC among first-degree relatives, cigarette smoking, and body mass index. Participants with calculated risk scores ≥ 4 were defined as high-risk (referred for colonoscopy), and those with scores < 4 were defined as low-risk (referred for FIT).

Table S1 Modified Asia-Pacific Colorectal Risk Score used in the TARGET-C trial

**Risk factor**	**Criteria**	**Points**
Age (years)	50 – 54	0
	55 – 64	1
	65 – 74	2
Sex	Women	0
	Men	1
Family history of colorectal cancer among first-degree relatives	Absent	0
	Present	1
Smoking	Non-smoker	0
	Current or past smoker	1
Body mass index (kg/m^2)	< 23	0
	≥ 23	1

Operation manual of the fecal immunochemical test (Pupu Tube®, New Horizon Health Technology Co., Ltd) used in the TARGET-C trial

【LIMITATION】

1.This test is qualitative and cannot be used to determine the concentration of human hemoglobin in the sample.

2.Due to the limitation of test principle, false negative result may appear with high dose sample (> 25mg/ml human hemoglobin). If high hemoglobin concentration is suspected and negative result is obtained, it is recommended to review other methods for questionable negative results.

【PERFORMANCE CHARACTERISTICS】

Sensitivity: This test kit can detect hemoglobin concentration above 100ng/mL.

Repeatability: The concordance rate is 100% when the test is performed on manufacturer's negative and positive reference standards.

Specificity:

The test kit does not cross-react with materials listed in the following table:

Item	Con.	Item	Con.
Pig hemoglobin	500μg/mL	Goat hemoglobin	500μg/mL
Bovine hemoglobin	500μg/mL	Rabbit hemoglobin	500μg/mL
Chicken hemoglobin	500μg/mL	Horseradish Peroxidase	2000μg/mL
Human myoglobin	1μg/mL		

Precision: The inter- and intra-CV of this test kit are both less than 10%.

【WARNINGS AND PRECAUTIONS】

1.For in vitro diagnostic use only.

2.Please read all the information in this leaflet before performing the test.

3.Do not use the kit after the expiry date in the label.

4.Do not use the kit if pouch is damaged.

5.Do not eat, drink or smoke in the area where the specimens or kits are handled.

6.Do not open the sealed foil pouch until you are ready to start the test.

7.As with all diagnostic tests, a final clinical diagnosis should not be based on the results of a single test, but should only be made by a physician after all clinical and lab findings have been evaluated.

8.Do not reuse the test strip. The used test kit should be discarded according to local regulations.

9.All fecal specimens and used strip should be considered potentially infectious, and contact with skin should be avoided.

10.High humidity and high temperature can adversely affect results.

【BIBLIOGRAPHY】

1.Simon J.B. Occult Blood Screening for Colorectal Carcinoma: A Critical Review,Gastroenterology, Vol. 1985; 88: 820.

2. Blebea J. and Mcpherson RA. False-Positive Guaiac Testing With Iodine, ArchPathol Lab Med, 1985;109:437-40.

3. Zheng Hongtu, Huang Yuping, Zhang Jingyan , Clinical use of Fecal Occult Blood Test kit, Chinese Journal of laboratory Diagnostics, 2011; 6

【MANUFACTURER】

HANGZHOU NEW HORIZON HEALTH TECHNOLOGY CO., LTD.

Room 1313, Floor 13, Building 2, No.400 Jiang Er Road, Changhe Street, Binjiang District, Hangzhou, Zhejiang, China, 310052.

Instruction Manual

Fecal Occult Blood Test Kit

Version: 1.0

【PRODUCT NAME】

Fecal Occult Blood Test Kit (Colloid Gold Immunoassay)

【PACKAGE】

One tube per pack

【INTENDED USE】

Fecal Occult Blood Test Kit (Colloid Gold Immunoassay) is used for the in-vitro qualitative detection of human occult blood in feces. It is used to aid in the diagnosis of gastrointestinal (GI) bleeding. The product is a single-use disposable in vitro diagnostic test, which can be self-administered by the end user.

Fecal occult blood (FOB) refers to blood in the feces that is not visibly apparent. Many diseases in the GI track can cause Fecal Occult Blood in the feces. Early stage conditions such as colon cancer, ulcers, polyps, colitis, diverticulitis, and fissures may be asymptomatic, except for the presence of occult blood in fecal samples. Therefore, through the detection of gastrointestinal bleeding, FOB testing can be effective to aid in the early detection and diagnosis of these conditions.

【PRINCIPLE】

Fecal Occult Blood Test Kit (Colloid Gold Immunoassay) utilizes double antibodies sandwich immunoassay for the detection of hemoglobin in fecal samples.

The test strip is pre-coated with anti-hemoglobin antibody on the test line region. During testing, the specimen reacts with the gold particle-labeled anti-hemoglobin antibody. The mixture migrates upward on the membrane chromatographically by capillary action to react with the second anti-hemo-globin antibody in the test line and generate a colored line in the test region. The presence of colored line in the test line region (T) indicates a positive result, while its absence indicates a negative result. To serve as a procedural control, a colored line will always appear in the control line region (C). If the control line does not appear, the test result is invalid.

【COMPOSITION】

Each pouch contains one test tube, which contains one test strip, 2ml of sample diluent, tube cap with the sampling stick, desiccant and the **Instruction Manual**.

The test strip contains two anti-hemoglobin antibodies, one of which is colloid gold labeled; anti-mouse IgG antibody; colloid gold-labeled mouse IgG and cellulose nitrate membrane.

Accessory required but not provided:

1. Clock or Timer
2. Dry and clean container used to collect feces.

【STORAGE AND STABILITY】

The sealed pouch should be stored at room temperature (2-30℃ or 35.6-86℉). The kit is stable within the expiry date printed on the label.

Do Not Freeze.

Once the pouch is opened, the test tube should be used within one hour. Prolonged exposure to hot and humid environment will cause product deterioration.

【SPECIMEN】

Collect stool into a clean, dry container, and sample stool in five different spots by using the sampling stick to complete sample collect (see illustration below).

Do Not let specimen come into contact with water or urine, and **do not** collect specimen with obvious presence of fresh blood.

Fresh stool should be used for testing. If not used immediately, stool should be stored at room temperature (2-30℃ or 35.6-86℉) for no more than 8 hours.

【DIRECTIONS FOR USE】

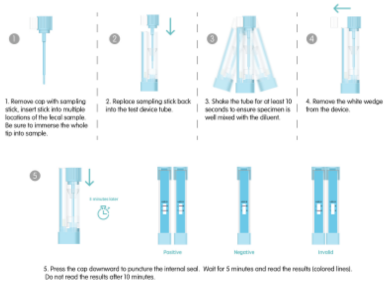

【INTERPRETATION OF RESULTS】

IMPORTANT: Test results **MUST** be interpreted within 5-10 minutes, after 10 minutes the result should be considered invalid.

POSITIVE*: Two colored lines appear. One line should be in the control line region (C) and another line should be in the test line region (T).

***NOTES:** The intensity of color in the test line region (T) will vary depending on the concentration of Fecal Occult Blood present in the specimen. Therefore, any shade of color in the test line region (T) **within 5-10 minutes** should be considered positive.

NEGATIVE:** One colored line appears in the control line region (C). No apparent colored line appears in the test line region (T).

****NOTES: Do Not read the strip after 10 minutes or more.** With prolonged time colored line may appear in the test line region (T) even for negative samples.

INVALID: No line appears in the control line region (C). If this occurs, read the directions again and repeat the test with a new test kit. If the result is still invalid, stop using the test kit immediately and contact your local distributor.

Study flow of the TARGET-C trial

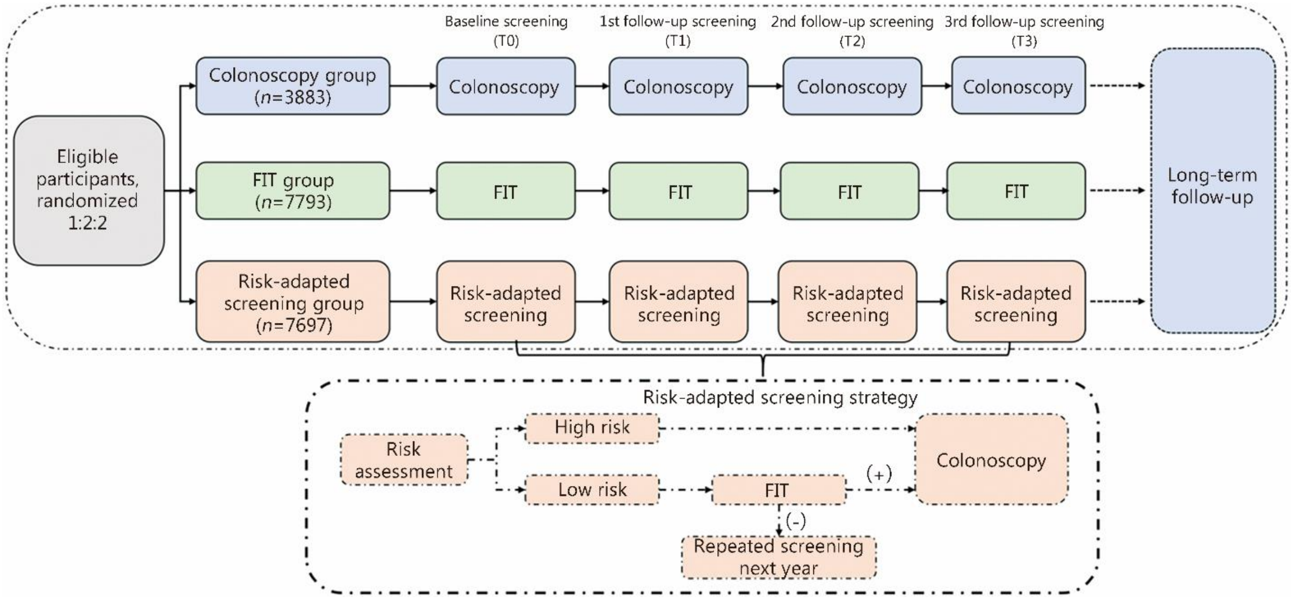

*Risk assessment was not conducted during the T1 phase, and the same risk stratification criteria used in the baseline phase were applied. ** Repeated risk assessments were conducted during the T2 phase, with the same risk stratification criteria applied in the T3 phase as in the T2 phase. FIT fecal immunochemical test

Detailed methods for costing estimation

Costing from a societal perspective

1) Direct medical cost: the per-person costs of risk assessment, colonoscopy examination, and biopsy were estimated using a micro-costing approach. Costs for FIT test and anesthesia (approximately 10% of the participants undertaking colonoscopy examination) were estimated based on the local medical service charging price [Chinese Yuan (CNY) 20.00 and CNY 450.00, respectively, reported by the study settings] and a cost-to-price ratio (approximately 0.76) reported by a previous micro-costing study [2].
2) Direct non-medical cost: questionnaire interview was administered among a total of 1586 participants, to evaluate the non-medical expenses during the whole screening process, such as the out-of-pocket spending on transportation.
3) Indirect cost: the same 1586 participants were also asked about how long the screening process took for themselves and their accompanied (if any). Using a human capital approach (CNY 90,501, the average salary of employees in urban non-private companies/institutions in 2019 in China) and the age-specific employment rates (reported by the Organization for Economic Co-operation and Development, 2020 for China), the obtained number of hours was then converted to CNY values.
4) Overhead cost: the management staff time, equipment, and supplies costs in relation to the project management, participants' recruitment, and callback, were collected by interviewing the project managers, reviewing the information in management documents, etc. In addition, a randomized controlled trial (RCT) usually costs more than a routine screening program, therefore, the value of preliminary aggregated overhead cost of the current RCT was then halved for base case analysis, the consequent proportion of overhead cost among the overall costs was more comparable to the median proportion (31.8%) of several previous studies included in a systematic review [3].
5) **Table S2** presents the aggregated costs (CNY) for each participant/procedure for imputing parameters.

Table S2 The aggregated costs for each participant (CNY), from a societal perspective

Category	Risk assessment	FIT test	Colonoscopy examination
Direct medical cost	1.94	15.20	259.02*
Direct non-medical cost	2.73	2.82	9.24
Indirect cost	76.01	65.16	399.11**

*The costs for biopsy (CNY 186.22) and for anesthesia (CNY 342.00) were separately estimated. **Considering the participants' restrictions/inability to work during preparation for colonoscopy and after colonoscopy, additional indirect costs were considered for the following items: 1) bowel preparation before the colonoscopy examination: assuming half day off needed (CNY 199.55); 2) anesthesia procedure: assuming 1.0 d off needed by an individual participant and 1.0 day off by his or her caregiver (CNY 798.22); 3) biopsy procedure: assuming 2.5 d off needed after the colonoscopy examination (CNY 997.77). Based on the main RCT data by screening arm and screening round, the parameters of the proportion of participants who received anesthesia and/or biopsy procedure(s) were applied in the indirect costing process. *CNY* Chinese Yuan, *FIT* fecal immunochemical test

Costing from a government perspective

In parallel, an analysis from a government perspective was also conducted using a package payment format.

1) The costs for the risk assessment and colonoscopy examination were informed by a large-scale ongoing screening program in China, and the cost for the FIT test was selected based on the findings of a systematic review (mostly qualitative testing) and generally a higher price for quantitative FIT testing [3].
2) To evaluate the robustness of the results from a government perspective, a range of sensitivity analyses were conducted, and more detailed reasons for value setting were presented in the table below.
3) **Table S3** presents the aggregated costs (CNY) for each participant/procedure.

Table S3 The aggregated costs for each participant (CNY), from a government perspective

**Perspectives and sub-categories**	**Risk assessment**	**FIT**	**Colonoscopy examination**
Base case	5.00	20.00	450.00[a]
Sensitivity analysis 1 (the lower limits gathered by a systematic review) [3]	0.08	1.80	300.00
Sensitivity analysis 2 (the upper limits gathered by a systematic review) [3]	2.00	9.00	568.00
Sensitivity analysis 3 (assumed)	15.00	50.00	NA

[a]The cost for the biopsy was included. *CNY* Chinese Yuan, *FIT* fecal immunochemical test, *NA* not available

Method 2

MIMIC-CRC overview

Overview

The Microsimulation Model for Prevention and Intervention of Colorectal Cancer in China (MIMIC-CRC) is a dynamic, individual-based model designed to simulate the natural history of colorectal cancer (CRC) in the Chinese population. It integrates demographic, epidemiological, clinical, and economic data to evaluate the impact of various screening strategies on CRC incidence, mortality, and cost-effectiveness. The framework of MIMIC-CRC is constructed to represent a synthetic cohort of the Chinese population, with each individual simulated independently. The model accounts for age, sex, and regional differences within China. Throughout their lifetimes, individuals are at risk of developing colorectal adenomas, which may progress to invasive cancer through defined pathways. The model also incorporates the effect of screening interventions, which can detect and lead to the removal of precancerous lesions or early-stage cancers, thus potentially preventing cancer progression. The model provides evidence and outcomes to guide policymakers in comparing and optimizing CRC screening strategies that maximize the use of scarce financial and organizational resources to save lives. In the current study, we aimed to compare alternative screening strategies to evaluate the long-term effectiveness and cost-effectiveness of three arms in the TARGTE-C trial.

Model structure

MIMIC-CRC simulates the development of CRC via the conventional adenoma-carcinoma pathway (over 95% in the Asian population) in the general average-risk population. Considering most individuals in the general population may develop at most one adenoma, the model simulates up to one adenoma per individual simultaneously. As each simulated person ages, the normal colon epithelium is at risk of developing non-advanced adenomas and advanced adenomas (≥ 10 mm in size, with villous components, or high-grade intraepithelial neoplasia). Advanced adenomas can progress to preclinical CRC (stages I-IV), which may develop into clinical (symptom-detected or screening-detected) CRC (stages I-IV). The figure below shows the model's natural history pathways.

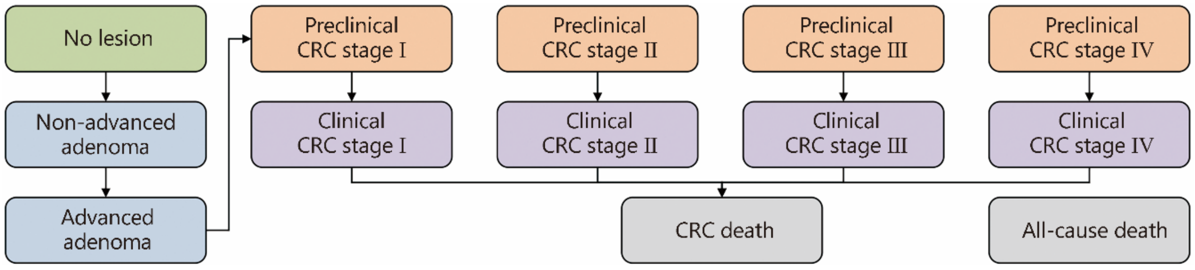

AA advanced adenoma, CRC colorectal cancer, NAA non-advanced adenoma.

Data sources

The data sources for MIMIC-CRC are diverse and comprehensive, ensuring the model is grounded in the best available evidence. Detailed parameters were shown in **Table S4**. Natural history parameters were obtained from several recent large-scale pooled analyses or high-quality meta-analyses of studies in China. When unavailable, we adapted parameters from well-established models included in the Cancer Intervention and Surveillance Modeling Network (CISNET). Population demographics were derived from the 2020 China Population Census, providing age, sex, and regional distributions. Cancer incidence and mortality rates were based on data from the China National Cancer Center and the Global Burden of Disease Study, adjusted to reflect regional variations. Screening and treatment data were informed by large-scale clinical trials conducted in China and internationally, as well as guidelines from the Chinese Ministry of Health and other expert bodies. Cost data were obtained from the latest studies, which include costs associated with CRC screening, diagnosis, treatment, and follow-up care.

Table S4 Detailed model parameters for natural history, utility, and cost used in MIMIC-CRC

**Parameters**	**Value**	**References**
**For baseline state distribution**		
Population proportion (sex-age-region-specific)	0.001 – 0.009	[4]
Proportion of clinical CRC	0.000 – 0.013	[5]
Stage of diagnosis (I; II; III; IV)	Male: 0.149; 0.333; 0.332; 0.186<br>Female: 0.157; 0.324; 0.341; 0.179	[6]
Ratio of preclinical/clinical CRC (I; II; III; IV)	(0.357 – 0.527) vs. (0.132 – 0.249);<br>(0.058 – 0.125) vs. (0.225 – 0.319)	[7]
Ratio of AA/CRC (sex-specific)	12	[8]
Ratio of NAA/CRC (sex-specific)	32	[8]
**For state transition**		
Annual progress rate from no-lesion to NAA	0.008	[9], Calibration[a]
Annual progress rate from NA to NAA	0.035	[9], Calibration[a]
Annual progress rate from AA to preclinical CRC stage I	0.020	[9], Calibration[a]
Annual progress rate from preclinical CRC stage I to preclinical CRC stage II	0.250	[9], Calibration[a]
Annual progress rate from preclinical CRC stage II to preclinical CRC stage III	0.300	[10], Calibration[a]
Annual progress rate from preclinical CRC stage III to preclinical CRC stage IV	0.350	[10], Calibration[a]
Symptom-detected rate of CRC stage I	0.146	[7]

Parameters	Value	References
Symptom-detected rate of CRC stage II	0.000	[7]
Symptom-detected rate of CRC stage III	0.040	[7]
Symptom-detected rate of CRC stage IV	0.070	[7]
Mortality rate (all-causes, sex-age-region-specific)	0.000 – 1.000	[4]
**Utility by stage**		
General population / NAA	50 – 54 years old: 0.957<br>55 – 59 years old: 0.955<br>60 – 64 years old: 0.957<br>≥ 65 years old: 0.943	[11]
AA	0.870	[11]
Stage I	0.742	[11]
Stage II	0.722	[11]
Stage III	0.756	[11]
Stage IV	0.745	[11]
**Treatment cost by CRC stage (CNY)**		
Stage I	56,099	[12]
Stage II	59,952	[12]
Stage III	67,292	[12]
Stage IV	82,729	[12]
**Other cost (CNY)**		
Pathological examination	1183.99	[13]
**Discounted rate**	0.050	-

AA advanced adenoma, *CRC* colorectal cancer, *CNY* Chinese Yuan, *NAA* non-advanced adenoma. [a]The parameter values were not only derived from the literature but were also adjusted to optimally fit our specific dataset.

Calibration process

The calibration of MIMIC-CRC involved adjusting model parameters to closely match observed epidemiological data on CRC incidence and mortality in China. The calibration process was divided into 7 steps: 1) Parameters to include in the calibration: starting parameter values were based on a literature review and expert opinion; 2) Selection of calibration targets: model projections of incidence, mortality, and stage distribution would be compared to those reported by the nationwide cancer registration. Cumulative cancer cases and deaths of each cancer site measured at 7.0 years and 10.8 years of follow-up would be compared against the cancer incidence and mortality observed from

the China Kadoorie Biobank (CKB) prospective cohort study. The survival rates after diagnosis would be compared to the observed net survival rates from 17 population-based cancer registration; 3) Goodness-of-Fit measures: for each iteration, the model's output was compared to target data, including age- and sex-specific CRC incidence and mortality rates, using a weighted sum of squared differences as the objective function; 4) Parameter search strategies (optimization): the process utilized the genetic algorithm, a probabilistic technique that iteratively adjusted parameters to minimize the difference between model predictions and observed data. A genetic algorithm is a heuristic search and optimization algorithm inspired by the principles of natural selection and genetics from the biological world. It belongs to the larger class of evolutionary algorithms, used for solving optimization and search problems where the solution space is large or the problem itself is too complex for traditional optimization methods; 5) Convergence criteria; 6) Stopping rule: the calibration process concluded once changes in the objective function fell below a predetermined threshold, indicating an optimal fit between the model predictions and the observed data; 7) Integrating the results of the calibration and the economic parameters.

To better evaluate the long-term effectiveness and cost-effectiveness of screening strategies in the TARGET-C trial, we need to recalibrate the natural history parameters to capture the characteristics of the population in the TARGET-C trial. To account for heterogeneous CRC risk among individuals, an additional risk-stratification variable was introduced, dividing participants into low-risk and high-risk groups. Calibration was performed using a simulated annealing algorithm to minimize discrepancies between observed and simulated data. This process involved two steps: first, calibrating the baseline risk distribution using baseline data from the TARGET-C study; second, calibrating the transition parameters for the low-risk and high-risk subgroups to reflect their distinct progression dynamics. Since most CRC risk factors predominantly influenced cancer initiation, we hypothesized that the transition rate from normal tissue to non-advanced adenoma varied between risk groups. This calibration was informed by data from four rounds of screening in the TARGET-C study. The final model effectively captured disease progression dynamics in both low- and high-risk populations, offering a robust microsimulation framework tailored to CRC risk stratification in the Chinese population.

Modelling analyses

The analysis adopted a cross-sectional approach to estimate year-by-year health outcomes, costs, and resource utilization for the population from the TARGET-C trial over 15 years. The cohorts were constructed to match the age and sex distributions of the population from the TARGET-C trial at recruitment. This analysis simulated the natural history of CRC in a fixed cohort of 200,000 individuals (10 times the original size of the population from TARGET-C). Screening frequency was defined as follows: 1) Colonoscopy: one-time screening within ages 50 – 74 years; 2)

FIT: annual screening for four consecutive rounds (years 1 – 4); 3) Risk-adapted screening: biennial risk assessment (APCS score) at T0 and T2 (years 1 and 3), with annual FIT for low-risk individuals (APCS < 4) and one-time colonoscopy for high-risk individuals (APCS ≥ 4). Screening eligibility was restricted to ages 50 – 74 years, consistent with TARGET-C inclusion criteria. Surveillance post-polyp removal followed 10-year colonoscopy intervals for individuals with prior negative colonoscopies. By projecting cross-sectional outcomes, this analysis provided insights into long-term trends in CRC incidence, mortality, costs, and resource requirements associated with different screening strategies and coverage levels. The primary outcomes were CRC incidence, CRC-related mortality, cost, and quality-adjusted life years (QALYs) of the different screening strategies compared with colonoscopy screening. The number needed to screen to prevent one CRC case was calculated by dividing the total number of FIT or colonoscopies used by the total number of CRC cases prevented during the study period. In this analysis, the study period was divided into three distinct periods: 5 years after start, 5 – 10 years, and 10 – 15 years. By segmenting the study period, we could observe the short-term, mid-term, and long-term impacts of different CRC screening strategies. We also calculated the incremental cost-effectiveness ratios by dividing the incremental costs by the incremental QALYs gained for each strategy and discounting them at a rate of 5%. We defined strategies with an incremental cost-effectiveness ratio of 0.5 to 2 times China's gross domestic product per capita (CNY 35,446 and 141,784) per QALYs gained as strongly and weakly cost-effective, respectively. The median of the results of 1000 iterations of the best parameter set was used to represent the point estimates, and 95% uncertainty intervals were used to quantify the uncertainty.

Sensitivity and probabilistic analyses

Univariate (one-way) and multivariate deterministic sensitivity analyses were conducted to assess the sensitivity of the results to changes in model input parameters, including utility, screening costs, diagnostic costs, discount rates, screening completion rates, screening sensitivity, and specificity. Additionally, we performed probabilistic sensitivity analyses under 1000 varied parameter sets to address joint uncertainties in the input parameter values.

References:

1. Lu M, Zhang YH, Lu B, Cai J, Liu CC, Chen HD, et al. Head-to-head comparison of the test performance of self-administered qualitative vs. laboratory-based quantitative fecal immunochemical tests in detecting colorectal neoplasm. Chin Med J (Engl). 2021;134(11):1335–44.
2. He SJ, Shi JF, Liao XZ, Zheng S, Zhu SL, Wang L. Micro-costing study of breast cancer intervention in China. HTAi Annual Meeting. Rome, Italy. 2017.
3. Wang H, Huang HY, Liu CC, Bai FZ, Zhu J, Wang L, et al. Health economic evidence for colorectal cancer screening programs in China: an update from 2009-2018. Zhonghua Liu Xing Bing Xue Za Zhi. 2020;41(3):429–35.
4. Chinese Population Censes Yearbook 2020. https://www.stats.gov.cn/sj/pcsj/rkpc/7rp/zk/indexce.htm. Accessed 10 Jan 2024.
5. Institute for Health Metrics and Evaluation. GBD Results. https://vizhub.healthdata.org/gbd-results. Accessed 10 Jan 2024.
6. Zeng H, Ran X, An L, Zheng R, Zhang S, Ji JS, et al. Disparities in stage at diagnosis for five common cancers in China: a multicentre, hospital-based, observational study. Lancet Public Health. 2021;6(12):e877–87.
7. Cardoso R, Guo F, Heisser T, De Schutter H, Van Damme N, Nilbert MC, et al. Proportion and stage distribution of screen-detected and non-screen-detected colorectal cancer in nine European countries: an international, population-based study. Lancet Gastroenterol Hepatol. 2022;7(8):711–23.
8. Chen H, Li N, Ren J, Feng X, Lyu Z, Wei L, et al. Participation and yield of a population-based colorectal cancer screening programme in China. Gut. 2019;68(8):1450–7.
9. Lu B, Wang L, Lu M, Zhang Y, Cai J, Luo C, et al. Microsimulation Model for Prevention and Intervention of Coloretal Cancer in China (MIMIC-CRC): development, calibration, validation, and application. Front Oncol. 2022;12:883401.
10. Ren Y, Zhao M, Zhou D, Xing Q, Gong F, Tang W. Cost-effectiveness analysis of colonoscopy and fecal immunochemical testing for colorectal cancer screening in China. Front Public Health. 2022;10:952378.
11. Chen H, Li N, Shi J, Ren J, Liu C, Zhang Y, et al. Comparative evaluation of novel screening strategies for colorectal cancer screening in China (TARGET-C): a study protocol for a multicentre randomised controlled trial. BMJ Open. 2019;9(4):e025935.
12. Huang HY, Shi JF, Guo LW, Bai YN, Liao XZ, Liu GX, et al. Expenditure and financial burden for the diagnosis and treatment of colorectal cancer in China: a hospital-based, multicenter, cross-sectional survey. Chin J Cancer. 2017;36(1):41.

13. Chen H, Shi J, Lu M, Li Y, Du L, Liao X, et al. Comparison of colonoscopy, fecal immunochemical test, and risk-adapted approach in a colorectal cancer screening trial (TARGET-C). Clin Gastroenterol Hepatol. 2023;21(3):808–18.
