# Supplementary material for: Effectiveness and cost-effectiveness of risk-adapted colorectal cancer screening: a randomized controlled trial and modeling analysis
Source: Mil Med Res. 2025 Nov 24;12:82. doi: 10.1186/s40779-025-00671-7 (PMC12642085; doi:10.1186/s40779-025-00671-7)
Supplement: Supplementary file 2 — Additional file 2. Table S1 Baseline study population characteristics among 3 screening arms [n (%)]. Table S2 Participation across the 3 screening arms from the T0 to T3 phases. Table S3 Participation of the risk-adapted screening arm from the T0 to T3 phases. Table S4 Cumulative participation across the 3 screening arms from the T0 to T3 phases. Table S5 Detection for advanced colorectal neoplasm among invited individuals in the colonoscopy arm, fecal immunochemical test arm, and the risk-adapted screening arm for the 4 screening rounds (ITT analysis). Table S6 Detection for any colorectal neoplasm among invited individuals in the colonoscopy arm, fecal immunochemical test arm, and the risk-adapted screening arm for the 4 screening rounds (ITT analysis). Table S7 Detected colorectal neoplasm of the included participants in the colonoscopy arm, fecal immunochemical test arm, and the risk-adapted screening arm for the cumulative 4 rounds of screening (PP analysis). Table S8 Number of colonoscopies needed to be performed to detect 1 neoplasm (ITT analysis) [number of colonoscopies (95% CI)]. Table S9 The costs per case detected, by trial arm and by screening round, from a societal perspective. Table S10 The costs per case detected, by trial arm and by screening round, from a government perspective. Table S11 Projected 5-, 10-, and 15-year incidence and mortality from the modeling analysis. Fig. S1 Detailed scheme of the study participants in the colonoscopy arm, fecal immunochemical test arm (FIT), and the risk-adapted screening arm from the T0 to T3 phases. Fig. S2 Cost-effectiveness analyses between screening arms under different scenarios with varying colonoscopy and fecal immunochemical test (FIT) adherence. Fig. S3 Result of the determined sensitivity analysis of colonoscopy screening compared to fecal immunochemical test screening (a) and risk-adapted screening (b). Fig. S4 Result of probabilistic sensitivity analysis. [file 40779_2025_671_MOESM2_ESM.pdf]

Table S1 Baseline study population characteristics among 3 screening arms [*n* (%)]

Factors	Screening arm			*P*-value [a]
	Colonoscopy (n = 3883)	FIT (n = 7793)	Risk-adapted screening (n = 7697)	
Sex				0.172
Men	1617 (41.6)	3310 (42.5)	3155 (41.0)	
Women	2266 (58.4)	4483 (57.5)	4542 (59.0)	
Age (years)				0.545
50 – 54	906 (23.3)	1825 (23.4)	1836 (23.9)	
55 – 59	830 (21.4)	1603 (20.6)	1574 (20.4)	
60 – 64	992 (25.5)	1924 (24.7)	1886 (24.5)	
65 – 69	807 (20.8)	1729 (22.2)	1658 (21.5)	
70 – 74	348 (9.0)	712 (9.1)	743 (9.7)	
Education background [b]				0.614
< High school	2701 (73.4)	5605 (72.2)	5595 (72.9)	
High school and equivalent	696 (18.9)	1542 (19.9)	1495 (19.5)	
Postsecondary graduate	281 (7.6)	621 (8.0)	582 (7.6)	
BMI (kg/m^{2}) [b]				0.614
< 23	1395 (37.9)	2872 (37.0)	2860 (37.3)	
≥ 23	2283 (62.1)	4896 (63.0)	4812 (62.7)	
Intake of NSAIDs [b]				0.983
No	3505 (95.3)	7402 (95.3)	7307 (95.2)	
Occasionally	100 (2.7)	202 (2.6)	202 (2.6)	
Regular	73 (2.0)	164 (2.1)	163 (2.1)	
Cigarette smoking [b]				0.646
Non-smoker	2978 (81.0)	6269 (80.7)	6154 (80.2)	
Past smoker	572 (15.6)	1217 (15.7)	1212 (15.8)	
Current smoker	128 (3.5)	282 (3.6)	306 (4.0)	
Alcohol drinking [b]				0.168
Never	2659 (72.3)	5722 (73.7)	5649 (73.6)	
Seldom	491 (13.3)	1047 (13.5)	983 (12.8)	
Regular	528 (14.4)	999 (12.9)	1040 (13.6)	
Family history of CRC among the first-degree relatives [b]				< 0.001
Yes	160 (4.4)	335 (4.3)	473 (6.2)	
No	3427 (93.2)	7277 (93.7)	7038 (91.7)	
Unknown	91 (2.5)	156 (2.0)	161 (2.1)	

[a] *P*-values calculated by the Chi-square test. [b] Detailed epidemiological questionnaire data were missing in 205 cases in the colonoscopy arm, 25 cases in the FIT arm, and 25 cases in the risk-adapted screening arm. *BMI* body mass index, *CRC* colorectal cancer, *FIT* fecal immunochemical test, *NSAIDs* nonsteroidal anti-inflammatory drugs

Table S2 Participation across the 3 screening arms from the T0 to T3 phases

Screening round	Colonoscopy arm (*n* = 3883)		FIT arm (*n* = 7793)		Risk-adapted screening arm (*n* = 7697)	
	Screened/ Invited participants	% (95% CI)	Screened/ Invited participants	% (95% CI)	Screened/ Invited participants	% (95% CI)
**Overall**						
T0	1644/3883	42.3 (40.8 – 43.9)	7326/7793	94.0 (93.5 – 94.5)	6557/7697	85.2 (84.4 – 86.0)
T1	-	-	6048/6968	86.8 (86.0 – 87.6)	4913/6352	77.3 (76.3 – 78.4)
T2	-	-	6113/6693	91.3 (90.6 – 92.0)	4623/6131	75.4 (74.3 – 76.5)
T3	-	-	5768/7172	80.4 (79.5 – 81.3)	4362/6883	63.4 (62.3 – 64.5)
**Men**						
T0	706/1617	43.7 (41.3 – 46.1)	3114/3310	94.1 (93.2 – 94.8)	2334/3155	74.0 (72.4 – 75.5)
T1	-	-	2523/2900	87.0 (85.7 – 88.2)	1432/2294	62.4 (60.4 – 64.4)
T2	-	-	2511/2753	91.2 (90.1 – 92.2)	1183/2194	53.9 (51.8 – 56.0)
T3	-	-	2361/2951	80.0 (78.5 – 81.4)	1093/2593	42.2 (40.2 – 44.1)
**Women**						
T0	938/2266	41.4 (39.4 – 43.4)	4212/4483	94.0 (93.2 – 94.6)	4223/4542	93.0 (92.2 – 93.7)
T1	-	-	3525/4068	86.7 (85.6 – 87.7)	3481/4058	85.8 (84.7 – 86.8)
T2	-	-	3601/3940	91.4 (90.5 – 92.2)	3440/3937	87.4 (86.3 – 88.4)
T3	-	-	3407/4221	80.7 (79.5 – 81.9)	3269/4290	76.2 (74.9 – 77.5)

CI confidence interval, *FIT* fecal immunochemical test

Table S3 Participation of the risk-adapted screening arm from the T0 to T3 phases

Screening round	High-risk individuals		Low-risk individuals	
	Screened/ Invited participants	% (95% CI)	Screened/ Invited participants	% (95% CI)
T0	712/1453	49.0 (46.4 – 51.6)	5845/6219	94.0 (93.4 – 94.6)
T1	47/737	6.4 (4.8 – 8.5)	4866/5615	86.7 (85.7 – 87.5)
T2	96/915	10.5 (8.6 – 12.7)	4527/5007	90.4 (89.6 – 91.2)
T3	43/1163	3.7 (2.7 – 5.0)	4319/5480	78.8 (77.7 – 79.9)

CI confidence interval

Table S4 Cumulative participation across the 3 screening arms from the T0 to T3 phases

Screening round	Colonoscopy arm (*n* = 3883)		FIT arm (*n* = 7793)		Risk-adapted screening arm (*n* = 7697)	
	Screened/ Invited participants	% (95% CI)	Screened/ Invited participants	% (95% CI)	Screened/ Invited participants	% (95% CI)
**Overall**						
T0	1644/3883	42.3 (40.8 – 43.9)	7326/7793	94.0 (93.5 – 94.5)	6557/7697	85.2 (84.4 – 86.0)
T0 – T1	1644/3883	42.3 (40.8 – 43.9)	7614/7793	97.7 (97.3 – 98.0)	6808/7697	88.5 (87.7 – 89.1)
T0 – T2	1644/3883	42.3 (40.8 – 43.9)	7740/7793	99.3 (99.1 – 99.5)	6865/7697	89.2 (88.5 – 89.9)
T0 – T3	1644/3883	42.3 (40.8 – 43.9)	7775/7793	99.8 (99.6 – 99.9)	7122/7697	92.5 (91.9 – 93.1)
**Men**						
T0	706/1617	43.7 (41.3 – 46.1)	3114/3310	94.1 (93.2 – 94.8)	2334/3155	74.0 (72.4 – 75.5)
T0 – T1	706/1617	43.7 (41.3 – 46.1)	3239/3310	97.9 (97.3 – 98.3)	2432/3155	77.1 (75.6 – 78.5)
T0 – T2	706/1617	43.7 (41.3 – 46.1)	3290/3310	99.4 (99.1 – 99.6)	2459/3155	77.9 (76.5 – 79.4)
T0 – T3	706/1617	43.7 (41.3 – 46.1)	3301/3310	99.7 (99.5 – 99.9)	2614/3155	82.9 (81.5 – 84.1)
**Women**						
T0	938/2266	41.4 (39.4 – 43.4)	4212/4483	94.0 (93.2 – 94.6)	4223/4542	93.0 (92.2 – 93.7)
T0 – T1	938/2266	41.4 (39.4 – 43.4)	4375/4483	97.6 (97.1 – 98.0)	4376/4542	96.3 (95.8 – 96.9)
T0 – T2	938/2266	41.4 (39.4 – 43.4)	4450/4483	99.3 (99.0 – 99.5)	4406/4542	97.0 (96.5 – 97.5)
T0 – T3	938/2266	41.4 (39.4 – 43.4)	4474/4483	99.8 (99.6 – 99.9)	4508/4542	99.3 (98.9 – 99.5)

CI confidence interval, *FIT* fecal immunochemical test

Table S5 Detection for advanced colorectal neoplasm among invited individuals in the colonoscopy arm, fecal immunochemical test arm, and the risk-adapted screening arm for the 4 screening rounds (ITT analysis)

Screening round	Advanced neoplasm [% (95% CI)]			Colonoscopy vs. FIT		Colonoscopy vs. Risk-adapted screening		Risk-adapted screening vs. FIT	
	Colonoscopy (*n* = 3883)	FIT (*n* = 7793)	Risk-adapted screening (*n* = 7697)	*OR* (95% CI) [c]	*P*-value	*OR* (95% CI) [c]	*P*-value	*OR* (95% CI) [c]	*P*-value
**T0**									
Overall	2.8 (2.3 – 3.3)	1.2 (0.9 – 1.4)	1.6 (1.4 – 2.0)	2.45 (1.84 – 3.26)	< 0.001	1.69 (1.29 – 2.20)	< 0.001	1.45 (1.11 – 1.92)	0.008
Proximal colon [a]	1.4 (1.1 – 1.8)	0.5 (0.4 – 0.7)	0.6 (0.5 – 0.8)	2.62 (1.74 – 3.97)	< 0.001	2.21 (1.49 – 3.28)	< 0.001	1.19 (0.79 – 1.82)	0.409
Distal colon/rectum [b]	1.7 (1.4 – 2.2)	0.8 (0.6 – 1.0)	1.2 (0.9 – 1.4)	2.19 (1.54 – 3.12)	< 0.001	1.46 (1.06 – 2.02)	0.021	1.49 (1.08 – 2.08)	0.017
Men	4.1 (3.2 – 5.2)	1.9 (1.5 – 2.4)	3.4 (2.8 – 4.0)	2.19 (1.53 – 3.12)	< 0.001	1.21 (0.88 – 1.66)	0.237	1.79 (1.31 – 2.48)	< 0.001
Women	1.8 (1.3 – 2.4)	0.6 (0.4 – 0.9)	0.5 (0.3 – 0.7)	3.01 (1.86 – 4.94)	< 0.001	4.09 (2.44 – 7.08)	< 0.001	0.74 (0.41 – 1.30)	0.298
**T0 – T1**									
Overall	2.8 (2.3 – 3.3)	1.7 (1.4 – 2.0)	1.9 (1.6 – 2.2)	1.68 (1.29 – 2.18)	< 0.001	1.45 (1.12 – 1.87)	0.004	1.15 (0.91 – 1.47)	0.245
Proximal colon [a]	1.4 (1.0 – 1.8)	0.7 (0.6 – 0.9)	0.7 (0.6 – 0.9)	1.91 (1.30 – 2.79)	0.001	1.89 (1.29 – 2.76)	0.001	1.01 (0.70 – 1.47)	0.941
Distal colon/rectum [b]	1.7 (1.4 – 2.2)	1.2 (0.9 – 1.4)	1.4 (1.1 – 1.6)	1.50 (1.08 – 2.06)	0.014	1.26 (0.92 – 1.72)	0.148	1.18 (0.88 – 1.57)	0.266
Men	4.1 (3.2 – 5.2)	2.6 (2.1 – 3.2)	3.6 (3.0 – 4.4)	1.54 (1.10 – 2.13)	0.011	1.11 (0.81 – 1.51)	0.514	1.38 (1.04 – 1.83)	0.029
Women	1.8 (1.3 – 2.4)	1.0 (0.7 – 1.3)	0.7 (0.5 – 1.0)	1.96 (1.27 – 3.02)	0.002	2.69 (1.69 – 4.32)	< 0.001	0.73 (0.46 – 1.16)	0.187

Screening round	Advanced neoplasm [% (95% CI)]			Colonoscopy vs. FIT		Colonoscopy vs. Risk-adapted screening		Risk-adapted screening vs. FIT	
	Colonoscopy (*n* = 3883)	FIT (*n* = 7793)	Risk-adapted screening (*n* = 7697)	*OR* (95% CI) [c]	*P*-value	*OR* (95% CI) [c]	*P*-value	*OR* (95% CI) [c]	*P*-value
**T0 – T2**									
Overall	2.8 (2.3 – 3.3)	2.2 (1.9 – 2.5)	2.4 (2.0 – 2.7)	1.30 (1.01 – 1.65)	0.037	1.19 (0.93 – 1.51)	0.156	1.09 (0.88 – 1.34)	0.440
Proximal colon [a]	1.4 (1.1 – 1.8)	1.0 (0.8 – 1.2)	0.9 (0.7 – 1.1)	1.46 (1.03 – 2.05)	0.031	1.56 (1.10 – 2.20)	0.012	0.94 (0.68 – 1.29)	0.681
Distal colon/rectum [b]	1.7 (1.4 – 2.2)	1.5 (1.2 – 1.8)	1.6 (1.4 – 2.0)	1.19 (0.88 – 1.61)	0.244	1.06 (0.79 – 1.42)	0.680	1.12 (0.87 – 1.44)	0.384
Men	4.1 (3.2 – 5.2)	3.3 (2.8 – 4.0)	4.2 (3.5 – 4.9)	1.17 (0.85 – 1.60)	0.320	0.96 (0.70 – 1.29)	0.785	1.22 (0.94 – 1.58)	0.132
Women	1.8 (1.3 – 2.5)	1.3 (1.0 – 1.7)	1.1 (0.8 – 1.5)	1.53 (1.03 – 2.27)	0.033	1.80 (1.20 – 2.70)	0.004	0.85 (0.58 – 1.24)	0.410
**T0 – T3**									
Overall	2.8 (2.3 – 3.3)	2.3 (2.0 – 2.6)	2.6 (2.3 – 3.0)	1.21 (0.95 – 1.55)	0.124	1.06 (0.83 – 1.34)	0.658	1.15 (0.93 –1.41)	0.197
Proximal colon [a]	1.4 (1.1 – 1.8)	1.2 (1.0 – 1.5)	1.3 (1.0 – 1.5)	1.28 (0.92 – 1.77)	0.133	1.26 (0.90 – 1.74)	0.170	1.02 (0.77 – 1.36)	0.883
Distal colon/rectum [b]	1.7 (1.4 – 2.2)	1.6 (1.4 – 1.9)	2.0 (1.7 – 2.3)	1.16 (0.86 – 1.56)	0.324	0.92 (0.69 – 1.22)	0.562	1.26 (0.99 – 1.61)	0.060
Men	4.1 (3.2 – 5.2)	3.5 (2.9 – 4.2)	4.5 (3.9 – 5.3)	1.14 (0.83 – 1.56)	0.409	0.88 (0.65 – 1.19)	0.409	1.30 (1.01 – 1.67)	0.046
Women	1.8 (1.3 – 2.5)	1.4 (1.1 – 1.8)	1.2 (1.0 – 1.6)	1.35 (0.90 – 2.01)	0.144	1.52 (1.00 – 2.28)	0.046	0.89 (0.62 – 1.28)	0.531

[a] including invitees who had advanced neoplasms located at the proximal colon only, and at both proximal colon and distal colon/rectum. [b] including invitees who had advanced neoplasms located at the distal colon/rectum only, and at both proximal colon and distal colon/rectum. [c] *ORs* and 95% CIs were adjusted for age, sex (not for subarm analysis of men and women), and participating center in the logistic regression models. *CI* confidence interval, *FIT* fecal immunochemical test, *ITT* intention-to-treat, *OR* odds ratio

Table S6 Detection for any colorectal neoplasm among invited individuals in the colonoscopy arm, fecal immunochemical test arm, and the risk-adapted screening arm for the 4 screening rounds (ITT analysis)

Screening round	Any colorectal neoplasm [% (95% CI)]			Colonoscopy vs. FIT		Colonoscopy vs. Risk-adapted screening		Risk-adapted screening vs. FIT	
	Colonoscopy (*n* = 3883)	FIT (*n* = 7793)	Risk-adapted screening (*n* = 7697)	*OR* (95% CI) [c]	*P*-value	*OR* (95% CI) [c]	*P*-value	*OR* (95% CI) [c]	*P*-value
**T0**									
Overall	10.3 (9.4 – 11.3)	3.3 (2.9 – 3.7)	4.9 (4.4 – 5.4)	3.53 (2.99 – 4.16)	< 0.001	2.35 (2.01 – 2.73)	< 0.001	1.54 (1.31 – 1.82)	< 0.001
Proximal colon [a]	5.0 (4.3 – 5.7)	1.5 (1.3 – 1.8)	2.2 (1.9 – 2.5)	3.43 (2.72 – 4.34)	< 0.001	2.41 (1.94 – 2.98)	< 0.001	1.45 (1.14 – 1.84)	0.002
Distal colon/rectum [b]	6.2 (5.4 – 7.0)	2.1 (1.8 – 2.5)	3.1 (2.7 – 3.5)	3.14 (2.55 – 3.86)	< 0.001	2.11 (1.75 – 2.55)	< 0.001	1.50 (1.23 – 1.84)	< 0.001
Men	14.4 (12.7 – 16.1)	5.0 (4.3 – 5.8)	9.4 (8.4 – 10.5)	3.25 (2.63 – 4.03)	< 0.001	1.64 (1.35 – 1.98)	< 0.001	1.98 (1.63 – 2.43)	< 0.001
Women	7.4 (6.4 – 8.6)	2.1 (1.7 – 2.5)	1.7 (1.4 – 2.2)	3.98 (3.07 – 5.19)	< 0.001	4.79 (3.65 – 6.34)	< 0.001	0.85 (0.62 – 1.15)	0.280
**T0 – T1**									
Overall	10.3 (9.4 – 11.3)	4.4 (4.0 – 4.9)	5.8 (5.3 – 6.4)	2.60 (2.23 – 3.03)	< 0.001	1.93 (1.67 – 2.23)	< 0.001	1.37 (1.18 – 1.59)	< 0.001
Proximal colon [a]	5.0 (4.3 – 5.7)	2.0 (1.8 – 2.4)	2.5 (2.2 – 2.9)	2.55 (2.06 – 3.17)	< 0.001	2.07 (1.68 – 2.55)	< 0.001	1.24 (1.00 – 1.54)	0.046
Distal colon/rectum [b]	6.2 (5.4 – 7.0)	2.8 (2.5 – 3.2)	3.8 (3.4 – 4.2)	2.31 (1.91 – 2.80)	< 0.001	1.69 (1.41 – 2.02)	< 0.001	1.38 (1.15 – 1.65)	0.001
Men	14.4 (12.7 – 16.1)	6.5 (5.7 – 7.4)	10.5 (9.5 – 11.6)	2.45 (2.00 – 3.00)	< 0.001	1.44 (1.20 – 1.74)	< 0.001	1.69 (1.41 – 2.03)	< 0.001
Women	7.4 (6.4 – 8.6)	2.9 (2.4 – 3.4)	2.6 (2.2 – 3.1)	2.82 (2.22 – 3.59)	< 0.001	3.19 (2.50 – 4.08)	< 0.001	0.90 (0.70 – 1.16)	0.420

Screening round	Any colorectal neoplasm [% (95% CI)]			Colonoscopy vs. FIT		Colonoscopy vs. Risk-adapted screening		Risk-adapted screening vs. FIT	
	Colonoscopy (*n* = 3883)	FIT (*n* = 7793)	Risk-adapted screening (*n* = 7697)	*OR* (95% CI) [c]	*P*-value	*OR* (95% CI) [c]	*P*-value	*OR* (95% CI) [c]	*P*-value
**T0 – T2**									
Overall	10.3 (9.4 – 11.3)	5.6 (5.1 – 6.1)	7.0 (6.4 – 7.5)	2.02 (1.75 – 2.34)	< 0.001	1.60 (1.39 – 1.84)	< 0.001	1.28 (1.12 – 1.47)	< 0.001
Proximal colon [a]	5.0 (4.3 – 5.7)	2.6 (2.3 – 3.0)	3.0 (2.6 – 3.4)	1.97 (1.61 – 2.41)	< 0.001	1.73 (1.42 – 2.11)	< 0.001	1.15 (0.95 – 1.39)	0.155
Distal colon/rectum [b]	6.2 (5.4 – 7.0)	3.5 (3.1 – 4.0)	4.4 (4.0 – 4.9)	1.84 (1.54 – 2.20)	< 0.001	1.43 (1.20 – 1.70)	< 0.001	1.29 (1.09 – 1.52)	0.003
Men	14.4 (12.7 – 16.1)	8.0 (7.1 – 9.0)	11.9 (10.8 – 13.1)	1.92 (1.58 – 2.33)	< 0.001	1.23 (1.03 – 1.48)	0.023	1.55 (1.31 – 1.84)	< 0.001
Women	7.4 (6.4 – 8.6)	3.8 (3.3 – 4.4)	3.5 (3.0 – 4.0)	2.17 (1.73 – 2.70)	< 0.001	2.40 (1.91 – 3.01)	< 0.001	0.91 (0.73 – 1.14)	0.429
**T0 – T3**									
Overall	10.3 (9.4 – 11.3)	5.9 (5.4 – 6.5)	7.5 (6.9 – 8.1)	1.89 (1.63 – 2.18)	< 0.001	1.45 (1.26 – 1.67)	< 0.001	1.32 (1.15 – 1.50)	< 0.001
Proximal colon [a]	5.0 (4.3 – 5.7)	3.0 (2.7 – 3.4)	4.0 (3.2 – 4.0)	1.75 (1.44 – 2.13)	< 0.001	1.46 (1.21 – 1.77)	< 0.001	1.20 (1.00 – 1.44)	0.047
Distal colon/rectum [b]	6.2 (5.4 – 7.0)	3.8 (3.4 – 4.2)	5.0 (4.6 – 5.6)	1.77 (1.48 – 2.11)	< 0.001	1.30 (1.09 – 1.53)	0.003	1.37 (1.17 – 1.60)	< 0.001
Men	14.4 (12.7 – 16.1)	8.5 (7.6 – 9.5)	12.8 (11.7 – 14.0)	1.82 (1.50 – 2.20)	< 0.001	1.13 (0.94 – 1.36)	0.176	1.60 (1.36 – 1.89)	< 0.001
Women	7.4 (6.4 – 8.6)	4.0 (3.5 – 4.7)	3.8 (3.3 – 4.4)	1.99 (1.59 – 2.48)	< 0.001	2.15 (1.71 – 2.69)	< 0.001	0.93 (0.75 – 1.16)	0.540

[a] including invitees who had advanced neoplasms located at the proximal colon only, and at both proximal colon and distal colon/rectum. [b] including invitees who had advanced neoplasms located at the distal colon/rectum only, and at both proximal colon and distal colon/rectum. [c] *ORs* and 95% CIs were adjusted for age, sex (not for subarm analysis of men and women), and participating center in the logistic regression models. *CI* confidence interval, *FIT* fecal immunochemical test, *ITT* intention-to-treat, *OR* odds ratio

Table S7 Detected colorectal neoplasm of the included participants in the colonoscopy arm, fecal immunochemical test arm and the risk-adapted screening arm for the cumulative 4 rounds of screening (PP analysis)

Colorectal neoplasm	Colonoscopy [*n* = 3883, *n* (%)]	FIT [*n* = 7793, *n* (%)]	Risk-adapted screening [*n* = 7697, *n* (%)]	Colonoscopy vs. FIT		Colonoscopy vs. risk-adapted screening		Risk-adapted screening vs. FIT	
				OR (95% CI) [a]	*P*-value	*OR* (95% CI) [a]	*P*-value	*OR* (95% CI) [a]	*P*-value
**Overall**									
Colorectal cancer	10 (0.6)	10 (0.1)	15 (0.2)	4.36 (1.76 – 10.79)	0.001	2.42 (1.04 – 5.41)	0.034	1.82 (0.83 – 4.21)	0.143
Advanced precancerous lesion	97 (5.9)	167 (2.2)	176 (2.5)	2.29 (1.75 – 2.97)	< 0.001	1.84 (1.41 – 2.39)	< 0.001	1.28 (1.03 – 1.60)	0.026
Non-advanced adenoma	293 (17.8)	283 (3.6)	359 (5.0)	5.49 (4.58 – 6.57)	< 0.001	3.49 (2.94 – 4.16)	< 0.001	1.58 (1.34 – 1.86)	< 0.001
Advanced neoplasm	107 (6.5)	177 (2.3)	191 (2.7)	2.42 (1.87 – 3.11)	< 0.001	1.90 (1.48 – 2.44)	< 0.001	1.32 (1.07 – 1.63)	0.011
Any neoplasm	400 (24.3)	460 (6.0)	550 (7.7)	4.83 (4.14 – 5.64)	< 0.001	3.33 (2.85 – 3.88)	< 0.001	1.52 (1.33 – 1.74)	< 0.001
**Advanced neoplasm**									
Proximal colon [b]	61 (3.7)	95 (1.2)	95 (1.3)	2.51 (1.79 – 3.49)	< 0.001	2.12 (1.51 – 2.95)	< 0.001	1.20 (0.90 – 1.61)	0.213
Distal colon/rectum [b]	73 (4.4)	125 (1.6)	147 (2.1)	2.20 (1.62 – 2.97)	< 0.001	1.61 (1.19 – 2.15)	0.002	1.44 (1.13 – 1.85)	0.003
Men	66 (9.4)	115 (3.5)	140 (5.4)	2.28 (1.64 – 3.14)	< 0.001	1.47 (1.07 – 2.00)	0.017	1.63 (1.26 – 2.11)	< 0.001
Women	41 (4.4)	62 (13.9)	51 (1.1)	2.71 (1.79 – 4.06)	< 0.001	3.00 (1.95 – 4.59)	< 0.001	0.85 (0.58 – 1.24)	0.404

Colorectal neoplasm	Colonoscopy [*n* = 3883, *n* (%)]	FIT [*n* = 7793, *n* (%)]	Risk-adapted screening [*n* = 7697, *n* (%)]	Colonoscopy vs. FIT		Colonoscopy vs. risk-adapted screening		Risk-adapted screening vs. FIT	
				OR (95% CI) [a]	*P*-value	*OR* (95% CI) [a]	*P*-value	*OR* (95% CI) [a]	*P*-value
**Any neoplasm**									
Proximal colon [b]	199 (12.1)	235 (3.0)	264 (3.7)	3.92 (3.19 – 4.80)	< 0.001	2.87 (2.34 – 3.51)	< 0.001	1.38 (1.15 – 1.66)	< 0.001
Distal colon/rectum [b]	249 (15.1)	296 (3.8)	371 (5.2)	4.07 (3.38 – 4.90)	< 0.001	2.65 (2.21 – 3.17)	< 0.001	1.57 (1.34 – 1.85)	< 0.001
Men	232 (32.9)	279 (8.5)	396 (15.2)	4.81 (3.91 – 5.93)	< 0.001	2.31 (1.89 – 2.82)	< 0.001	2.11 (1.78 – 2.50)	< 0.001
Women	168 (17.9)	181 (4.1)	154 (3.4)	4.86 (3.86 – 6.12)	< 0.001	5.43 (4.27 – 6.90)	< 0.001	0.88 (0.71 – 1.10)	0.263

[a] *OR* and 95% confidence intervals were adjusted for age, sex (not for subarm analysis of men and women), and participating center in the logistic regression models. [b] The sum may exceed the total number because of the possibility of findings in both the proximal and distal colon/rectum. *CI* confidence interval, *FIT* fecal immunochemical test, *OR* odds ratio, *PP* per-protocol

Table S8 Number of colonoscopies needed to be performed to detect 1 neoplasm (ITT analysis) [*n* (95% CI)]

Screening round	Advanced neoplasm			Any neoplasm		
	**Colonoscopy (*n* = 3883)**	**FIT (*n* = 7793)**	**Risk-adapted screening (*n* = 7697)**	**Colonoscopy (*n* = 3883)**	**FIT (*n* = 7793)**	**Risk-adapted screening (*n* = 7697)**
**T0**						
Overall	15.4 (12.8 – 18.5)	9.1 (7.5 – 11.1)	10.3 (8.8 – 12.2)	4.1 (3.8 – 4.5)	3.2 (2.9 – 3.5)	3.5 (3.2 – 3.8)
Men	10.7 (8.5 – 13.5)	6.5 (5.2 – 8.3)	8.0 (6.7 – 9.5)	3.0 (2.7 – 3.4)	2.5 (2.2 – 2.8)	2.8 (2.6 – 3.1)
Women	22.9 (17.0 – 30.9)	14.7 (10.4 – 21.1)	22.4 (14.9 – 34.0)	5.6 (4.9 – 6.4)	4.5 (3.8 – 5.4)	5.9 (4.9 – 7.3)
**T0 – T1**						
Overall	15.4 (12.8 – 18.5)	8.3 (7.1 – 9.7)	10.5 (9.0 – 12.2)	4.1 (3.8 – 4.5)	3.1 (2.9 – 3.4)	3.4 (3.2 – 3.7)
Men	10.7 (8.5 – 13.5)	6.2 (5.1 – 7.5)	8.2 (6.9 – 9.8)	3.0 (2.7 – 3.4)	2.5 (2.3 – 2.8)	2.9 (2.6 – 3.1)
Women	22.9 (17.0 – 30.9)	12.5 (9.4 – 16.7)	18.6 (13.4 – 26.1)	5.6 (4.9 – 6.4)	4.2 (3.6 – 4.9)	5.1 (4.3 – 6.0)
**T0 – T2**						
Overall	15.4 (12.8 – 18.5)	7.8 (6.8 – 9.0)	10.2 (8.9 – 11.8)	4.1 (3.8 – 4.5)	3.0 (2.8 – 3.3)	3.5 (3.2 – 3.7)
Men	10.7 (8.5 – 13.5)	5.9 (5.0 – 7.0)	8.3 (7.1 – 9.8)	3.0 (2.7 – 3.4)	2.5 (2.3 – 2.7)	2.9 (2.7 – 3.1)
Women	22.9 (17.0 – 30.9)	11.4 (8.9 – 14.5)	15.3 (11.8 – 20.1)	5.6 (4.9 – 6.4)	3.9 (3.5 – 4.5)	4.9 (4.3 – 5.6)
**T0 – T3**						
Overall	15.4 (12.8 – 18.5)	7.9 (6.9 – 9.1)	9.3 (8.2 – 10.7)	4.1 (3.8 – 4.5)	3.0 (2.8 – 3.3)	3.2 (3.0 – 3.4)
Men	10.7 (8.5 – 13.5)	5.9 (5.0 – 7.0)	7.8 (6.7 – 9.2)	3.0 (2.7 – 3.4)	2.4 (2.2 – 2.7)	2.8 (2.6 – 3.0)
Women	22.9 (17.0 – 30.9)	11.6 (9.1 – 14.9)	13.1 (10.1 – 17.0)	5.6 (4.9 – 6.4)	4.0 (3.5 – 4.5)	4.3 (3.7 – 4.9)

CI confidence interval, *FIT* fecal immunochemical test, *ITT* intention-to-treat

Table S9 The costs per case detected, by trial arm and by screening round, from a societal perspective

Screening round	Colonoscopy screening		FIT screening		Risk-adapted screening	
	n	Cost per case (CNY)	*n*	Cost per case (CNY)	*n*	Cost per case (CNY)
**T0**						
CRC	9	182,389	7	209,867	13	183,007
Advanced precancerous lesion	98	16,750	83	17,700	114	20,869
Non-advanced adenoma	293	5602	166	8850	249	9555
Advanced neoplasm	107	15,341	90	16,323	127	18,733
Any neoplasm	400	4104	256	5739	376	6327
**T0 – T1**						
CRC	9	182,389	8	302,690	14	229,169
Advanced precancerous lesion	98	16,750	122	19,849	133	24,123
Non-advanced adenoma	293	5602	213	11,369	302	10,624
Advanced neoplasm	107	15,341	130	18,627	147	21,826
Any neoplasm	400	4104	343	7060	449	7146
**T0 – T2**						
CRC	9	182,389	8	402,192	15	293,694
Advanced precancerous lesion	98	16,750	161	19,985	166	26,539
Non-advanced adenoma	293	5602	265	12,142	352	12,515
Advanced neoplasm	107	15,341	169	19,039	181	24,339
Any neoplasm	400	4104	434	7414	518	8505
**T0 – T3**						
CRC	9	182,389	10	391,591	15	335,336
Advanced precancerous lesion	98	16,750	170	23,035	192	26,198
Non-advanced adenoma	293	5602	282	13,886	397	12,670
Advanced neoplasm	107	15,341	180	21,754	207	24,300
Any neoplasm	400	4104	462	8476	604	8328

CNY Chinese Yuan, *CRC* colorectal cancer, *FIT* fecal immunochemical test

Table S10 The costs per case detected, by trial arm and by screening round, from a government perspective

Screening round	Colonoscopy screening		FIT screening		Risk-adapted screening	
	n	Cost per case (CNY)	*n*	Cost per case (CNY)	*n*	Cost per case (CNY)
**T0**						
CRC	9	82,200	7	73,520	13	57,393
Advanced precancerous lesion	98	7549	83	6200	114	6545
Non-advanced adenoma	293	2525	166	3100	249	2996
Advanced neoplasm	107	6914	90	5718	127	5875
Any neoplasm	400	1850	256	2010	376	1984
**T0 – T1**						
CRC	9	82,200	8	93,963	14	67,606
Advanced precancerous lesion	98	7549	122	6161	133	7116
Non-advanced adenoma	293	2525	213	3529	302	3134
Advanced neoplasm	107	6914	130	5782	147	6439
Any neoplasm	400	1850	343	2192	449	2108
**T0 – T2**						
CRC	9	82,200	8	122,970	15	80777
Advanced precancerous lesion	98	7549	161	6110	166	7299
Non-advanced adenoma	293	2525	265	3712	352	3442
Advanced neoplasm	107	6914	169	5821	181	6694
Any neoplasm	400	1850	434	2267	518	2339
**T0 – T3**						
CRC	9	82,200	10	113,738	15	90,960
Advanced precancerous lesion	98	7549	170	6690	192	7106
Non-advanced adenoma	293	2525	282	4033	397	3437
Advanced neoplasm	107	6914	180	6313	207	6589
Any neoplasm	400	1850	462	2462	604	2259

CNY Chinese Yuan, *CRC* colorectal cancer, *FIT* fecal immunochemical test

Table S11 Projected 5-, 10-, and 15-year incidence and mortality from the modeling analysis

Year after start	Screening strategy	Scenario [a]	Accumulated incidence per 100,000 persons	Percentage change of incidence compared with no screening	Accumulated mortality per 100,000 persons	Percentage change of mortality compared with no screening
5	No screening	-	282	-	65	-
	Colonoscopy screening	Status quo	239	-15.25%	46	-29.23%
		100%	181	-35.82%	46	-29.23%
	FIT screening	Status quo	325	15.25%	60	-7.69%
		100%	318	12.77%	54	-16.82%
	Risk-adapted screening	Status quo	284	0.71%	64	-1.54%
		100%	244	-13.48%	57	-12.31%
10	No screening	-	762	-	150	-
	Colonoscopy screening	Status quo	577	-24.28%	114	-24.00%
		100%	383	-49.74%	79	-47.33%
	FIT screening	Status quo	636	-16.54%	112	-25.33%
		100%	635	-16.67%	109	-27.33%
	Risk-adapted screening	Status quo	606	-20.47%	119	-20.67%
		100%	456	-40.16%	104	-30.67%
15	No screening	-	1552	-	298	-
	Colonoscopy screening	Status quo	1171	-24.55%	224	-24.83%
		100%	915	-41.04%	163	-45.30%
	FIT screening	Status quo	1325	-14.63%	210	-29.53%

Year after start	Screening strategy	Scenario [a]	Accumulated incidence per 100,000 persons	Percentage change of incidence compared with no screening	Accumulated mortality per 100,000 persons	Percentage change of mortality compared with no screening
		100%	1290	-16.88%	222	-25.50%
	Risk-adapted screening	Status quo	1293	-16.69%	234	-21.48%
		100%	964	-37.89%	191	-35.91%

[a] The participation rate of status quo in colonoscopy screening group is 42.34%; the participation rate of status quo in FIT screening group is 94.62%; the participation rate of status quo in risk-adapted screening group is 49.00% for high-risk group and 94.60% for low-risk group. *FIT* fecal immunochemical test, *QALY* quality-adjusted life year

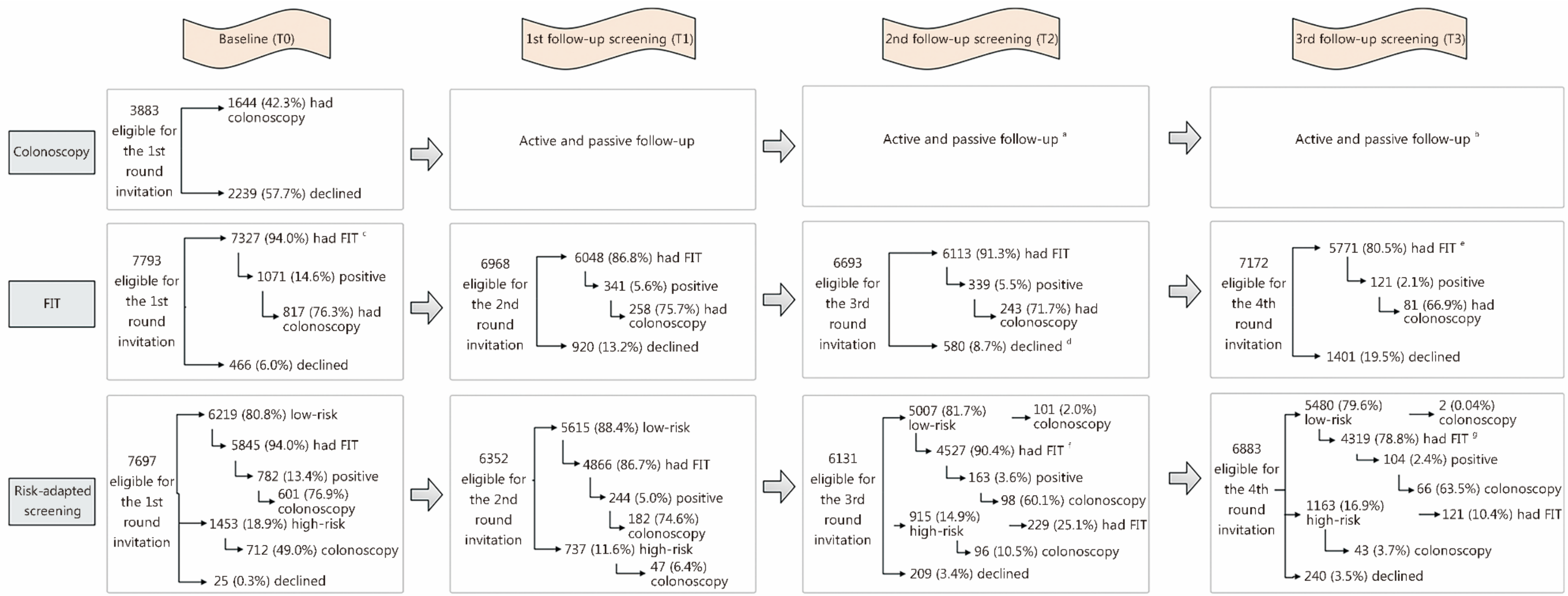

Fig. S1 Detailed scheme of the study participants in the colonoscopy arm, fecal immunochemical test arm (FIT), and the risk-adapted screening arm from the T0 to T3 phases. High- and low-risk were defined according to the Asia-Pacific Colorectal Screening (APCS) score: participants with a score ≥ 4 were classified as high-risk (referred for colonoscopy), and those with a score < 4 were classified as low-risk (referred for FIT). [a] 2 had a colonoscopy, and 8 had FIT at the third round of screening. [b] 1 had a colonoscopy, and 14 had FIT. [c] 1 had a colonoscopy with a negative FIT result. [d] 1 had a colonoscopy without having FIT. [e] 3 had a colonoscopy with negative FIT results. [f] 9 had colonoscopy with negative FIT results; [g] 2 had colonoscopy with negative FIT results

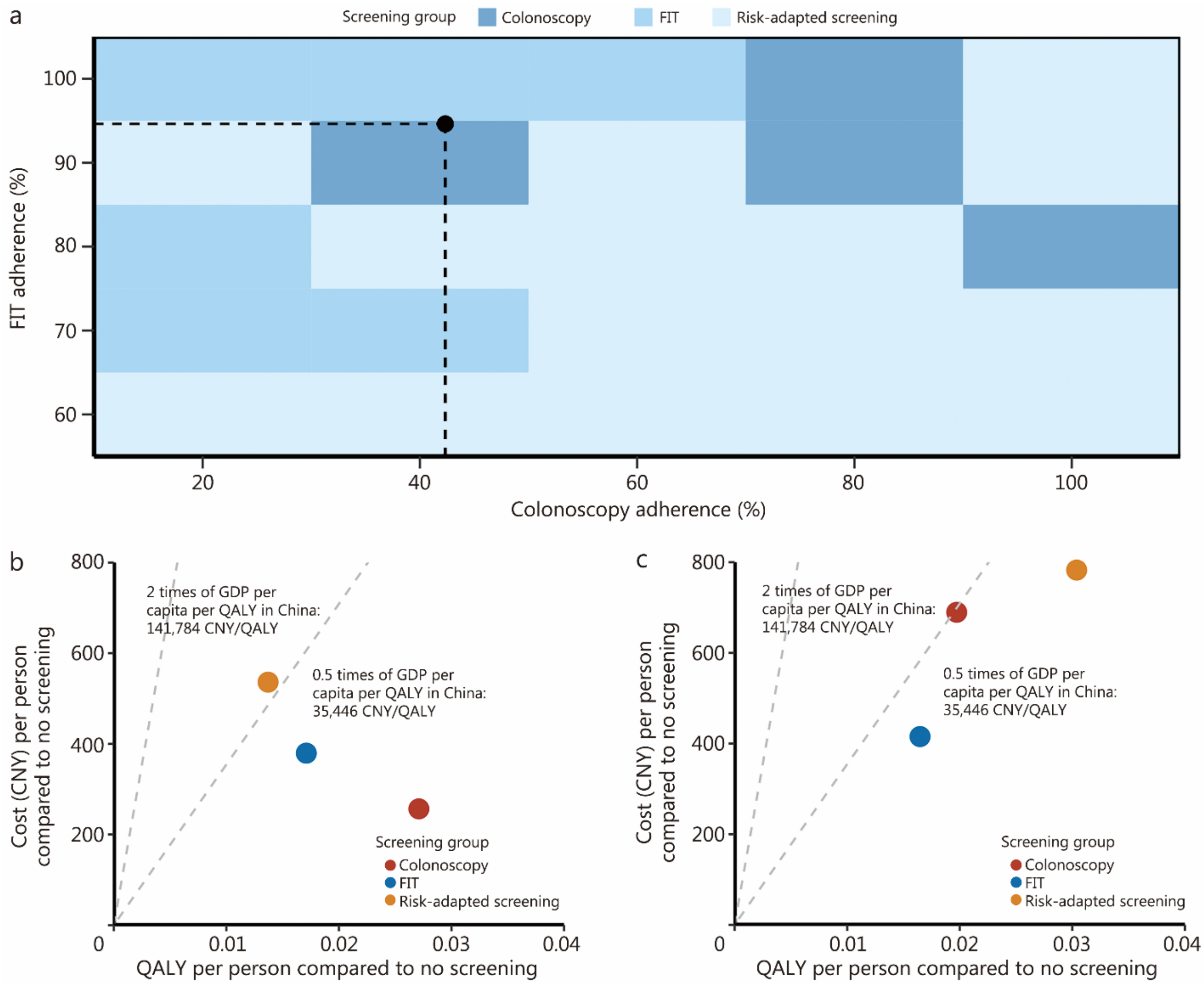

Fig. S2 Cost-effectiveness analyses between screening arms under different scenarios with varying colonoscopy and fecal immunochemical test (FIT) adherence. **a** Most cost-effective screening arm under different scenarios. **b** Cost-effectiveness plane plot under the current scenario. **c** Cost-effectiveness plane plot under 100% adherence scenario. CNY Chinese Yuan, GDP gross national product, QALY quality-adjusted life year

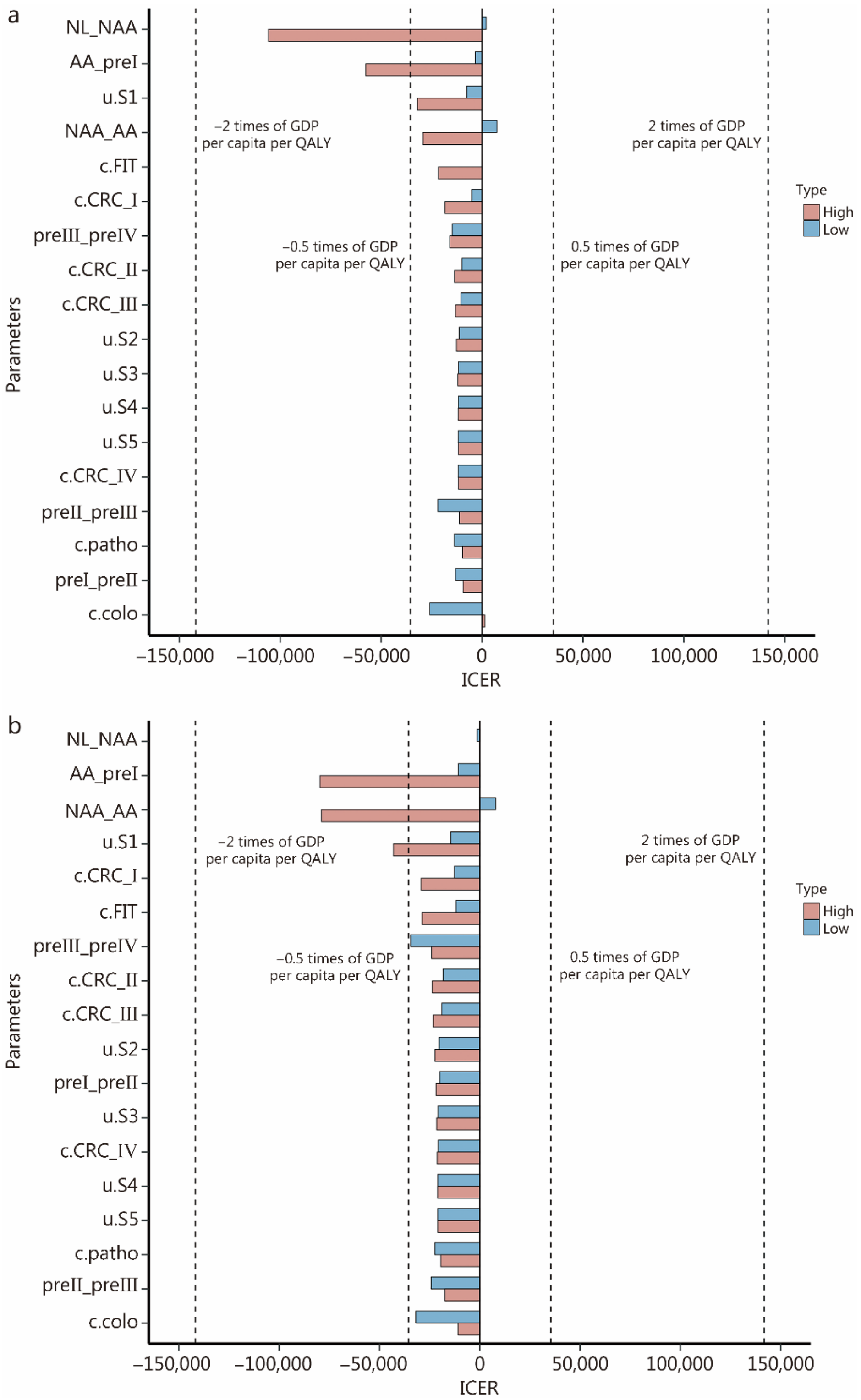

Fig. S3 Result of determined sensitivity analysis of colonoscopy screening compared to fecal immunochemical test screening (**a**) and risk-adapted screening (**b**). The type of high and low indicated the upper and lower bounds of the parameter range used in the sensitivity analysis. AA_preI = transition rate from advanced adenoma to preclinical CRC stage I, c.colo = cost of colonoscopy, c.FIT = cost of FIT, c.patho = cost of pathology, c.CRC_I = cost of treatment for CRC stage I, c.CRC_II = cost of treatment for CRC stage II, c.CRC_III = cost of treatment for CRC stage III, c.CRC_IV = cost of treatment for CRC stage IV, NL_NAA = transition rate from no lesion to non-advanced adenoma, NAA_AA = transition rate from non-advanced adenoma to advanced adenoma, preI_preII = transition rate from preclinical CRC stage I to preclinical CRC stage II, preII_preIII = transition rate from

preclinical CRC stage II to preclinical CRC stage III, preIII_preIV = transition rate from preclinical CRC stage III to preclinical CRC stage IV, u.S1 = utility of advanced adenoma, u.S2 = utility of CRC stage I, u.S3 = utility of CRC stage II, u.S4 = utility of CRC stage III, u.S5 = utility of CRC stage IV. GDP gross national product, ICER incremental cost-effectiveness ratio, QALY quality-adjusted life year

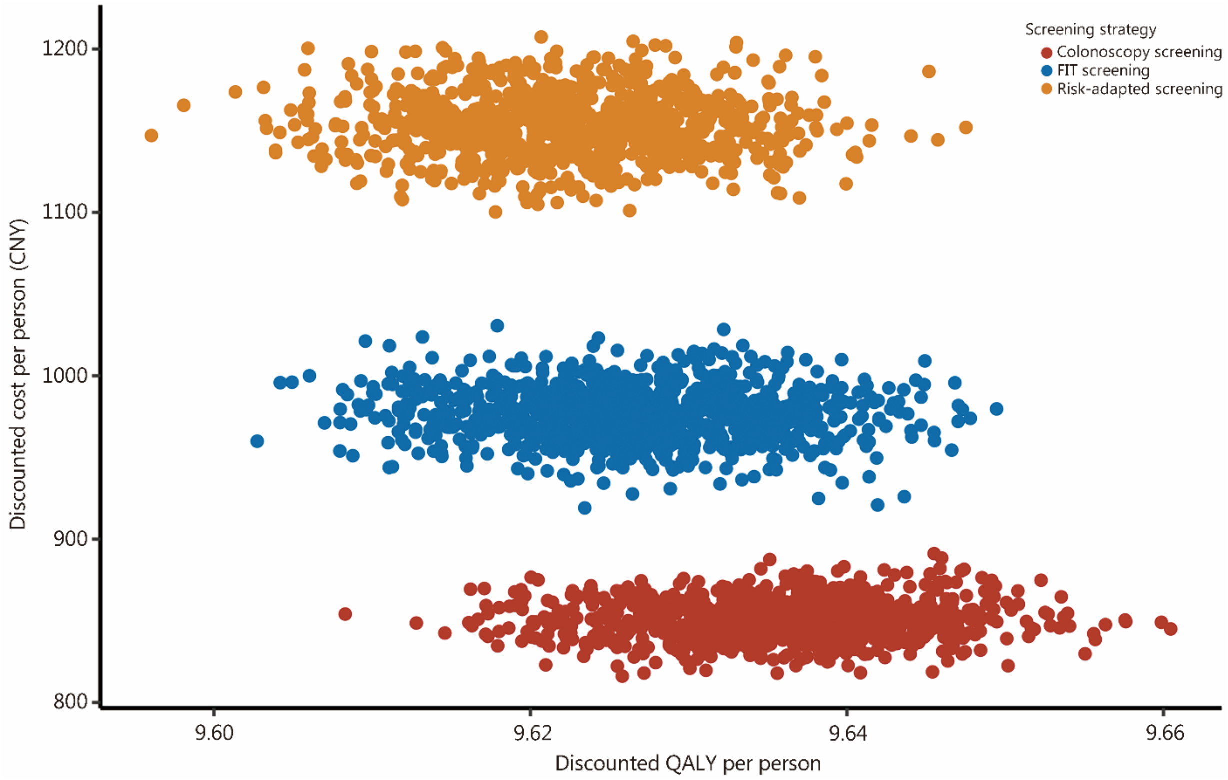

Fig. S4 Result of probabilistic sensitivity analysis. CNY Chinese Yuan, FIT fecal immunochemical test arm, QALY quality-adjusted life year
